# The processes and impacts of co-designed health interventions by and for Pacific populations: a scoping review

**DOI:** 10.1186/s12889-025-23795-w

**Published:** 2025-07-26

**Authors:** Siobhan Tu’akoi, Samuela ‘Ofanoa, Malakai ‘Ofanoa, Hinamaha Lutui, Maryann Heather, Felicity Goodyear-Smith

**Affiliations:** 1 Pacific Health section, School of Population Health, University of Auckland, Auckland, New Zealand; 2 The Cause Collective, Auckland, New Zealand; 3 Etu Pasifika Limited, Auckland, New Zealand; 4 Department of General Practice and Primary Health Care, University of Auckland, Auckland, New Zealand

**Keywords:** Co-design, Community-based, Participatory, Pacific, Pasifika, Health, Review

## Abstract

**Background:**

Globally, Pacific peoples face significant inequities across a range of health issues. While it is increasingly recognised that Pacific communities should be partners in the development of health interventions intended to benefit them, less is understood about the effectiveness or outcomes of these processes. This scoping review aimed to explore how health interventions co-designed by Pacific communities are defined, undertaken and the health outcomes that they achieve.

**Methods:**

This review was guided by the Joanna Briggs Institute methodology for scoping reviews and the Preferred Reporting Items for Systematic Reviews and Meta-Analyses extension for Scoping Reviews guidelines. Inclusion criteria were studies using participatory approaches with Pacific communities to develop health-related interventions and reported findings. Five electronic databases (Medline, Embase, Scopus, PsycINFO and CINAHL) were searched, along citation searching of reference lists and grey literature sources. Database results were imported into Covidence for independent eligibility screening by two reviewers. Critical appraisal and risk of bias were assessed for the final studies using the Joanna Briggs Institute checklists.

**Results:**

A total of 6972 records were identified, with 21 final studies included after screening. Studies were conducted across nine different countries on a range of health issues, with diabetes prevention or management being the most common. Fifteen different terms outlining participatory methodologies were found across the 21 studies, including community-based participatory research, empowerment, participatory approaches, action research and co-design. Although definitions and descriptions of participatory processes were often lacking, in general, studies found that interventions reported positive impacts on health-related outcomes for Pacific people.

**Conclusions:**

This review highlighted the process behind participatory research designs with Pacific communities and how these can result in positive health outcomes over time. Future research and intervention development in this area will benefit from clearer definitions and explanations of processes, strong evaluation structures implemented from the outset and longer timeframes for observation.

**Supplementary Information:**

The online version contains supplementary material available at 10.1186/s12889-025-23795-w.

## Background

In general, the purpose of health research is to build the knowledge base, inform best practices and treatments, and develop effective strategies for improving the health and well-being of populations. Despite the potential of health research to achieve these outcomes and reduce health disparities, on critique, research can often be deemed irrelevant for end-users or uninformed by local or cultural perspectives [1]. As a result, institutions and researchers are increasingly acknowledging the benefits of ensuring local communities are involved in or leading the research process to ensure relevancy and reduce research waste [2, 3]. Methodologies such as community-based participatory research (CBPR), co-design and participatory action research are based on partnerships with stakeholders, patients, and community members that are often used in health research for this purpose [4]. While these approaches are generally favoured for ensuring research is meaningful and has impact for the intended communities, studies continue to assert that there is a lack of evidence for effectiveness. Slattery and colleagues conducted a rapid overview of reviews focused on research co-design methods in health and found that while the approach appeared to be widely used, descriptions and evaluations of it were lacking [3]. Similarly, King et al. reviewed co-design theories utilised with Indigenous children and other young people from priority social groups, finding a lack of quality reporting regarding definitions, theory, and praxis [5]. Both reviews suggested that a greater level of detail and formal evaluation was required for co-design research projects moving forward.

Pacific people or ‘Pasifika’ is a collective term used typically in Western Countries to represent the ethnicity of people originating from Pacific Islands across Polynesia, Melanesia, and Micronesia [6]. Over time, communities from these island nations have increasingly migrated to larger Western countries such as Australia, Aotearoa New Zealand (NZ), and the United States [7]. In NZ, for example, Pacific peoples make up about 8% of the population, with 33.6% being born overseas [8]. Although typically representing smaller proportions of the population when outside their home countries, Pacific people are affected by significant health inequities related to non-communicable diseases, infectious diseases, and premature mortality, as a result of wider socioeconomic and environmental determinants [9]. While Pacific countries each have their own unique histories, traditions, and languages, values of family, collectivism, spirituality, and reciprocity are commonly held and differ from individualistic Western ideals [10]. This has impacts on the delivery of health care, where face-to-face engagement, shared decision-making and family approaches are often preferred [11].

In order to work towards improved health outcomes for Pacific people, who disproportionately experience health inequities, active participation by these groups in health interventions is critical [10]. A study in NZ evaluated five co-design projects conducted with Pacific and Indigenous Māori communities to qualitatively assess the challenges, solutions and lessons with these approaches [12]. They found that when authentic co-design was present, that is, greater power sharing and transparency with communities, significant benefits resulted, including increased trust, improved recruitment and retention of participants and capacity building [12]. Although similar studies acknowledge such benefits for the research process of equity and community ownership [13], less is understood in the literature about the outcomes of these approaches on Pacific people’s health. In order to map the existing evidence of co-design approaches with Pacific people and understand the breadth of intervention processes and outcomes, a scoping review was undertaken. The aim of this review was therefore to explore how health interventions co-designed by Pacific communities have been conducted and what is known about the health-related outcomes they have achieved.

The specific review objectives were:

1. To map existing studies on co-designed health interventions for Pacific population.
2. To examine how co-design or participatory methods are defined in health research with Pacific people.
3. To explore what is known about the outcomes of co-design and participatory research methodologies for improving Pacific people’s health.

## Methods

This scoping review was guided by the Joanna Briggs Institute (JBI) methodology for scoping reviews and followed the Preferred Reporting Items for Systematic Reviews and Meta-Analyses extension for Scoping Reviews (PRISMA-ScR) reporting guidelines [14].

### Eligibility criteria

Studies which reported on health outcomes of co-designed, health-related interventions with Pacific populations were sought for this review. Inclusions and exclusions were assessed by the JBI criteria of population/participants, concept and context [15]. The population of interest (P) were the Pacific population defined as those with ancestry to Pacific Island countries across Polynesia, Melanesia, and Micronesia (see Additional file 2: Search strategy for full list of countries). The concept (C) was co-designed health interventions, and the context (C) was global. For inclusion, there needed to be clear evidence of a partnership or participatory methodology whereby the interventions were either designed or co-designed by Pacific community members. Studies which additionally included non-Pacific people as the community partner in the design phase of interventions were excluded to ensure approaches were by Pacific, for Pacific, acknowledging the distinct culture and traditions that unite Pacific countries. Evidence of measuring a health-related outcome or finding as a result of the intervention was also required, for example, changes in metabolic health, dietary changes or screening uptake. Protocol papers, reviews and articles published in languages other than English were also excluded.

### Information sources and search strategy

Five databases were searched for relevant articles on 2 March 2023: Medline, Embase (both accessed via Ovid), Scopus, PsycINFO and CINAHL. Each search comprised synonyms across three main concepts: co-design method, health, and Pacific. The co-design concept included terms such as participatory, action research, co-create, co-produce, co-construct, and human-centred design. The health concept included variations of the terms ‘health’ and ‘wellbeing’. The Pacific concept included terms such as Pasifika, Melanesia, Micronesia, Polynesia, Tonga, Samoa, Hawaii, Cook Islands, Niue, and Fiji. The Boolean operator AND was used between concepts and OR within groups. Each database was searched per database requirements with the aid of a university librarian and MESH terms were included where appropriate. No filters or search limits were applied on the databases. Full search strategies, including all synonyms for each database, are available in Additional file 2: Search strategy. The references lists from final articles also underwent backward citation searching to identify any further relevant papers for inclusion. Grey literature sources, including governmental and health ministry websites, thesis repositories and organisation reports, were also searched using combinations of keywords from the three main concepts outlined above. Examples of grey literature sources included the World Health Organization, Pacific Community webpage, Google and ProQuest Thesis Repository (see Additional File 2 for full list). The database search strategy was re-run on 27 January 2025 to check for any updated articles and reports in the time since article submission, and grey literature searches were completed on 12 May 2025.

### Selection of sources of evidence

Database results and grey literature were exported into Covidence, a web-based review management software, and duplicates were removed. Titles and abstracts of articles were screened independently using the inclusion criteria by ST and SO. Studies that could not have eligibility confirmed were held for full text review. Full texts were then retrieved for the remaining articles and assessed for eligibility independently by ST and SO, with FGS available for adjudication when required. An agreement level of 86% was initially reached during screening and all conflicts were then resolved through discussion and consensus. Articles were excluded if the full text was unable to be retrieved.

### Data charting and synthesis of results

Data items from the final articles were extracted in Covidence by ST and SO including author, year, country, study design, nature of the co-design/participatory process, definition of method, Pacific population characteristics, health issue studied, details of the intervention, cultural considerations, participants involved in the evaluation and study findings/outcomes. No further information was requested from study authors beyond the scope of the articles. Due to the broad topic scope and heterogeneity of data, data from the final studies were analysed and described using textual narrative synthesis. This approach allowed for the reporting of study characteristics, context and quality, while exploring the differences and similarities between studies [16]. Basic descriptive statistics to summarise study characteristics were conducted on Excel.

The final studies in this review were appraised for risk of bias by one reviewer (SO) using the JBI critical appraisal tools [17] and cross-checked by ST. JBI checklists enabled the assessment of a range of different study types, including randomised controlled trials, quasi-experimental studies, qualitative research and narrative evidence. Studies were scored and assessed as high quality (70–100%), medium quality (50–69%) or low quality (0–49%) [18]. As a scoping review investigating the breadth of participatory research interventions undertaken with Pacific people, studies were not excluded based on quality if they fit the eligibility criteria for this review.

## Results

**Fig. 1:**
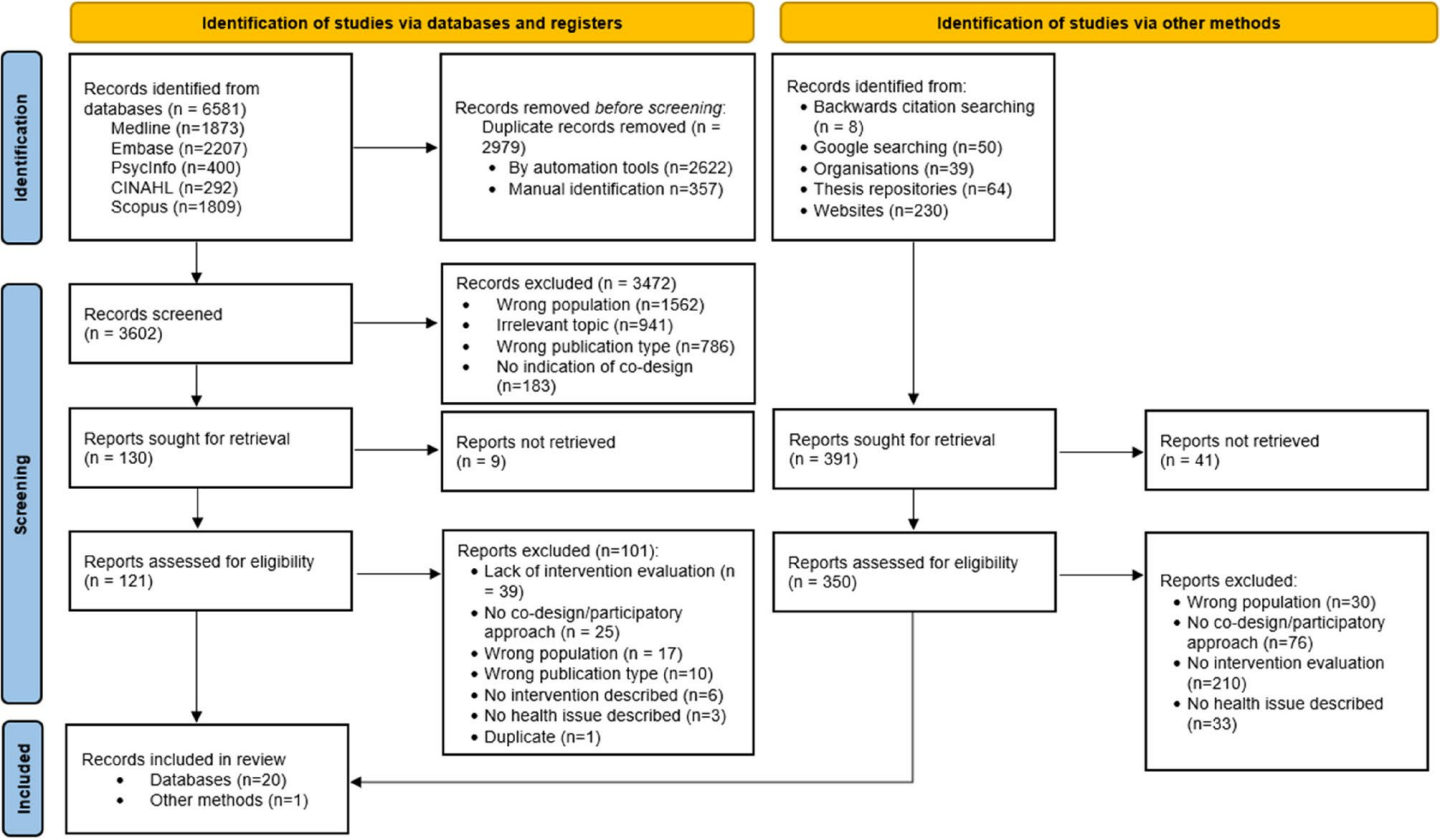
PRISMA-ScR flow diagram of included studies

A total of 6972 records were identified, 6581 via database searching and an additional 391 reports by other methods, as shown in Fig. 1. Duplicates were removed by automation and manual searching (*n* = 2979). The titles and abstracts of 3602 records were then independently screened by two reviewers ST and SO, with 3472 excluded. Full text eligibility was independently conducted on the remaining records (121 identified via databases and 350 by other methods), after 50 full texts were unable to be retrieved. A final total of 21 records were included in this review, including one paper identified for inclusion through backward citation searching. All 21 papers were appraised using the JBI tools, with majority of studies being rated of a high quality. Full results and scoring are available in Additional file 3: Quality assessment.

### Characteristics of included studies

Studies in this review ranged in publication date between 1994 and 2024, with most (71%) published within the last 15 years [19–33]. Geographically, studies were conducted across a range of countries, but primarily in high-income, Western settings. Eight studies were conducted in the United States [19, 20, 24, 27, 28, 32–34], four in NZ [21, 23, 30, 35], three in Australia [29, 31, 36], two in Papua New Guinea [37, 38] and one in each of American Samoa [39], Federated States of Micronesia [25], Fiji [26] and Tonga [22]. Regarding study design, eleven used a quasi or non-randomised experimental study designs [19–22, 25–29, 34, 35], four studies each used randomised controlled trials [24, 32, 33, 39] and qualitative approaches, respectively [23, 30, 31, 36] and two were case studies [37, 38].

### Participatory methods utilised with Pacific people

**Fig. 2:**
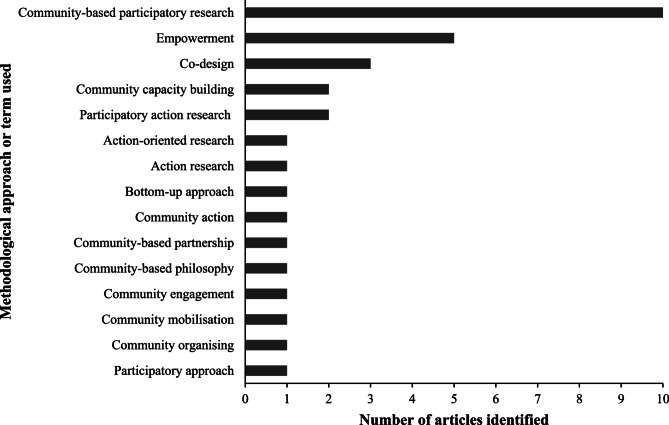
Types of methodological terms identified in included articles. Note: Some articles did not outline any methodological terms, while others identified multiple

A range of methodological approaches and terms were used, as highlighted in Fig. 2. Fifteen different terms were identified, with one high quality case study mentioning up to five different approaches [38]. CBPR was the most common approach acknowledged across the studies with 48% of articles (six high quality and four medium quality studies) [19, 20, 24, 27–30, 32, 33, 39], followed by empowerment (four high quality and one lower quality) [21, 30, 34, 37, 39], co-design (three high quality studies) [21, 29, 30] and participatory action research (two high quality studies) [21, 38]. Less common terms identified included ‘bottom-up approach’ and ‘community engagement’.

Sixty two percent of articles (*n* = 13) provided definitions for the methodologies used, all highlighting the importance of strong collaborations or partnerships with Pacific community members and ensuring community ownership throughout the duration of the research project and beyond [19, 21–23, 25, 26, 28–30, 36–39]. For example, a high quality pre-post study by McElfish and colleagues defined CBPR as a way to “engage non-traditional partners and honour their unique contributions at all phases of the research process from prioritising the research needs to disseminating the findings” [19, 21–23, 26, 28, 30, 38]. In discussing the use of an empowerment approach, Firestone and colleagues described that it could provide “a unique opportunity for community and researcher partnerships to be established, with a view of progressing a long-term collaboration to develop an in-depth reservoir of knowledge and capacity building” [21, p. 3]]. Definitions also outlined the potential of such approaches to engage underrepresented groups, address health inequities and ensure research projects are relevant to the communities intended to benefit. Nine studies, most of which were medium or low quality (*n* = 7), did not explicitly provide a definition for the methodology used [20, 24, 25, 27, 31–35], although some did cite their own or others’ previous research.

**Fig. 3:**
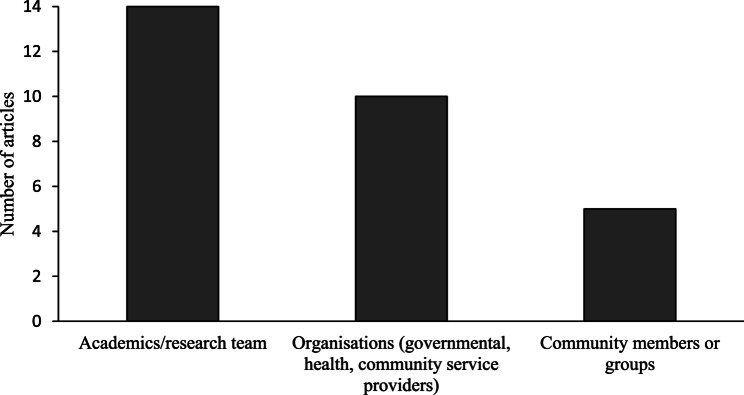
Groups identified as starting the co-design partnership. Note: Some studies identify more than one group

While all studies involved Pacific communities and stakeholders in the designing process of health interventions in some capacity, the level of detail regarding group composition, the nature of collaborations and the frequency of meetings varied. As shown in Fig. 3, most collaborative research partnerships were started, at least in part, by the research team (*n* = 14), followed by organisations and service providers (*n* = 10). While five studies noted that community members or groups were part of the project establishment in some way, only two specified that Pacific communities solely initiated the process. Both studies were of high quality, with one case study of a malaria prevention project in Papua New Guinea reporting the leading role of the Kewapi languages group [37] and one non-randomised controlled study in South Auckland, NZ, outlining how Pacific churches invited diabetes researchers to develop prevention programmes for members [35]. Most studies did not provide in-depth detail on the number of community members involved in the participatory process (57% of articles, *n* = 12), and of those that did, collaborations with smaller groups under 10 people were most common. Only two studies reported the age range of community partners, one high quality pre-post interventional study focused on Pacific youth in NZ aged 15–24 years [21] and another high quality non-randomised experimental study involving Micronesian women in Hawaii aged 22–69 years [19].

The way in which participatory processes were conducted with Pacific people was linked to specific cultural factors in nine studies [19, 21, 28–30, 35–37, 39]. Two papers outlined how empowering discussion-based techniques with Pacific community groups was culturally relevant and linked back to oral traditions and collective decision-making [35, 39] while one included the use of the Pacific Fonofale model as a guiding framework [30]. Oliver and colleagues conducted an action research approach with Cook Island community members in New South Wales, Australia, enabling separate groups for males and females to respect cultural protocols and allow open discussions for developing physical activity and nutrition interventions [36]. Focus group discussions were started with food and observed traditions such as prayer and songs to open and close sessions [36]. Discussion sessions to co-design interventions across the final studies were often assisted by Pacific translators, leaders or facilitators who had knowledge or a connection with the group and could converse in a specific Pacific language [19, 21, 35, 37]. Participatory approaches were identified as being particularly appropriate for research with Pacific communities due to prior evidence of success and their aim of reducing health inequities [28, 29] (Table 1).

**Table 1:** Summary table of included studies

Author/Year	Participatory method(s) stated	Pacific co-design partners	Aim of study	Health issue	Main outcome	Intervention	Evaluation participants	Main findings of co-designed intervention
Aitaoto 2012 [19]	Community-based participatory research	16 Micronesian women (aged 22–69).	To develop and track the reach of Micronesian women lay educators in implementing a cancer awareness program among Micronesian women living in Hawaii.	Breast cancer prevention	Increase in mammograms	Culturally tailored educational materials and toolkit, to be delivered by lay educators.	567 Micronesian women aged 18–75 years (202 women aged 40 or older eligible for mammography screening).	• 11 Micronesian lay educators provided cancer information sessions to 567 Micronesian women. • Among the 202 women aged 40 or older eligible for mammography screening, 166 (82%) had never had a mammogram and were assisted to appointments. • After 6 months, 146 (88%) of the 166 had received a mammogram, increasing compliance from 18–90%.
Chung-Do 2024 [20]	Community-based participatory research	Community leaders and residents of Waimānalo	To test the feasibility and acceptability of the MALAMA program as a public health intervention to promote healthy eating.	Food insecurity	Eating habits	Seven workshops: Indigenous knowledge, gardening and learning how to build and maintain home backyard aquaponics.	21 participants from 10 Native Hawaiian families enrolled in the MALAMA study (18–68 years).	• Fruit consumption among all participants significantly increased from time 1 (2.1 servings) to time 2 (2.9 servings). • No statistically significant differences in body mass index and waist-to-hip ratio were observed between any time points among participants, although there were favourable trends in blood pressure and fish and vegetable consumption.
Firestone 2021 [21]	Community-based partnership Participatory action research Empowerment Co-design	41 young Pasifika youth (15–24 years) from an Auckland health provider and a rural Waikato health provider	To co-design a small-scale community-based intervention, led by the Pasifika youth and implement and evaluate the short-term success of the interventions.	Diabetes prevention	Daily physical activity (measured by weight loss and step counts)	Eight-week programme with education on physical activity, nutrition and building health literacy.	Pacific and Māori aged between 25–44 years old with a high risk of developing prediabetes	• Significant positive changes, as evident by the mean percent change in weight loss (2.43%), mean percent change in waist circumference reduction (1.58%) and total average number of steps (range: 14,817 − 80,182 steps) accumulated from the start of the intervention (*p* < 0.001).
Fitzpatrick 2007 [37]	Empowerment	Members of the Kewapi language group living in urban Erima, Port Moresby	To explore how an empowerment approach could affect changes in malaria prevalence among remote members of the Kewapi language group in PNG.	Malaria prevention	Malaria-related deaths	Distribution of insecticide impregnated bed nets.	Community members in Batri village	• Records prior to November 2004 at the remote Batri Village Aid Post showed that, among the village population of 1400, there were five deaths from malaria each year and 29 consultations for malaria-related symptoms. Since the deployment of the nets in Nov 2004 there were no deaths from cerebral malaria in the community.
Fotu 2011 [22]	Community capacity building	Tongan community members, youth leaders, ministers, parents, town officers and health representatives.	To present the results of the Ma’alahi Youth Project, the first community-based intervention to target adolescent obesity in the Kingdom of Tonga.	Obesity	Body fat %	Social marketing, community capacity building and grass roots activities to promote healthy behaviours.	1083 adolescents in intervention communities. 1396 adolescents in the comparison group.	• No statistically significant differences in outcomes in weight, BMI and BMI-z, or prevalence of overweight/obesity between the intervention and comparison groups. • A small relative decrease in body fat in the intervention group (−1.5%, *P* < 0.0001). No other difference for any anthropometric variables between groups.
Han 2015 [23]	Community organising	A Pacific youth as a full-time lead organiser and a leadership team of five Pacific youths.	To improve the mental health outcomes of low-income minority youth by engaging them in community organizing.	Mental health and wellbeing	Mental health outcomes	Community organising campaign led and run by Pacific youth	Pacific youths	• Qualitative and quantitative data showed that youths experienced improvements in their mental health status. • Data from the surveys (*p* < 0.1) showed that youths felt calmer and more relaxed, reporting being more likely to think before acting and less likely to experience headaches, stomach aches or sickness.
Kaholokula 2017 [24]	Community-based participatory research	A kumu hula (hula expert) and two Native Hawaiian Pacific Islander community leaders	To test both feasibility and efficacy of Ola Hou in reducing the blood pressure in a community sample of Native Hawaiian Pacific Islanders with physician-diagnosed hypertension.	Hypertension	Blood pressure	12-week hula-based intervention with education on blood pressure management	55 participants with diagnosed hypertension	• Ola Hou participants, compared to wait-list control participants, had greater reductions in their systolic blood pressure in both the intention-to-treat analysis (−18.3 vs. −7.6 mmHg, respectively) and the complete case analysis (−19.8 vs. −9.2 mmHg, respectively) from baseline to 3-month assessment. • No significant differences in diastolic blood pressure between the intervention and control after 3 months.
Katz 2007 [38]	Action-oriented Participatory action Community mobilisation Capacity building Bottom-up approach	Papua New Guinean (PNG) “change agents” and local communities	To describe the Tingim Laip intervention, the participatory monitoring and evaluation mechanisms, and some of the preliminary findings.	HIV/AIDS	Attitudinal and behavioural change to prevent HIV	Tingim Lap intervention: peer education, community mobilisation, life skills development and sports/music interventions.	PNG people living with HIV/AIDS and youth	• 34 Tingim Laip sites around PNG reached an estimated at + 170,000 persons. • Qualitative data showed improvements as a result of the intervention and local STI services in most of the sites reported increased number of clients utilizing their services as a result of the demand generated by the intervention.
Kaufer 2010 [25]	Participatory approach	Mand community leaders and residents	To assess changes in diet and health that may have been impacted by the two-year intervention.	Non-communicable diseases	Dietary changes	Two-year food-based intervention focused on education and agricultural activities.	Random sample of households (*n* = 47) out of all 71 households in Mand.	• The average household diet in 2007 (follow-up) had significantly higher micronutrient intake, increased consumption frequency of promoted foods and greater dietary diversity than in 2005 (baseline). • Results indicated increased (110%) provitamin A carotenoid intake; increased frequency of consumption of local banana (53%), giant swamp taro (475%), and local vegetables (130%); and increased dietary diversity from local food.
Kremer 2011 [26]	Community capacity building	Fijian schools, students, teachers, community members, local council representative	To report on the outcomes of a 3-year obesity prevention study, Healthy Youth Healthy Communities undertaken with Fijian adolescents	Obesity	BMI & body fat %	Social marking, nutrition and physical activity initiatives.	7 intervention schools (*n* = 874) and 11 comparison schools (*n* = 2,062). Participants were aged 13–18 years.	• At follow-up, no differences for weight, body size and weight status classification. The intervention group had lower percentage body fat (−1.17). • The (unadjusted) proportion of children who reported having snack food every day after school was lower at follow-up for both groups. A positive (healthful) change in time spent watching TV was also observed.
McElfish 2015 [27]	Community-based participatory research	Marshallese community A Marshallese community leader and health worker on study team.	To use a community-based participatory approach to pilot test a family model of diabetes education conducted in participants homes with extended family members.	Diabetes	Glycemic control measured by A1C	10 h of diabetes education over six weekly sessions.	Six Marshallese families (27 participants).	• 78% of participants were retained in the study. Post-test results indicated a 5% reduction in A1C across all participants and a 7% reduction in those with type 2 diabetes.
McElfish 2019 [28]	Community-based participatory research	9 Marshallese community members and health professionals	To develop and test a health education video focused on blood glucose monitoring and control.	Type 2 diabetes	Participants’ self-efficacy related to glucometer usage	Health education video highlighting the importance of performing blood glucose checks.	50 Marshallese participants with type 2 diabetes mellitus (20 of which completed semi-structured interviews).	• Participants reported significant increases in self-efficacy related to glucometer use and the importance of performing blood glucose checks (*p* < 0.001) and a 1.45% reduction in A1C between preintervention and 12 weeks postintervention (*p* = 0.006). • Qualitative results indicated the video was both culturally appropriate and effective.
Mishra 2009 [39]	Community-based participatory research Empowerment	Samoan women	To test the effectiveness of a theory-guided, culturally tailored cervical cancer education program designed to increase Pap smear use.	Cervical cancer education	Utilisation of Pap smears	Cervical cancer education programme: education booklets, skill building, interactive group discussion sessions.	416 eligible women recruited from the 26 Samoan churches in the two study locations	• 120 women (30.2%) self-reported obtaining a Pap smear between the pretest and posttest surveys. Women in the intervention group (61.7%, *n* = 74, *p* < 0.01) compared to those in the control group (38.3%, *n* = 46) were significantly more likely to self-report having obtained a Pap smear.
Ndwiga 2020 [29]	Community-based participatory research Co-design	Samoan community representative reference group	To evaluate the effectiveness of a culturally adapted, church-based lifestyle intervention among Australian Samoans living in Sydney.	Type 2 diabetes	HbA1c	Church-based lifestyle education and support programme.	Three churches took part. 107/159 (67%) church attendees participated in the baseline.	• HbA1c dropped significantly between baseline and follow-up among participants with known diabetes (8.1 ± 2.4% (65 mmol/mol) vs. 7.4 ± 1.8% (57 mmol/mol); *p* = 0.040) and non-significantly among participants with newly diagnosed diabetes (8.0 ± 2.1% (64 mmol/mol) vs. 7.1 ± 2.3 (54 mmol/mol); *p* = 0.131). • There were no significant reductions in blood pressure, BMI or waist circumference at follow-up.
Oliver 2007 [36]	Action research	24 Cook Islanders in the Illawarra region (13 women and 11 men)	To develop appropriate interventions to increase physical activity rates and knowledge of nutrition.	Type 2 diabetes	Physical activity, nutrition	Lifestyle interventions: Walking groups, line dancing, gym work, food diaries and a nutrition workshop.	The same 24 Cook Islands participants who were involved in developing the interventions.	• After six weeks, members of the walking groups were still walking regularly, some having increased activity from three times per week to daily. • Some women had lost a considerable amount of weight, which motivated others to increase their exercise levels.
Prapaveissis 2022 [30]	Community-based participatory research Co-design Empowerment	Pacific health providers and community partners	To investigate empowerment and co-design modules to build the capacity of Pasifika youth to develop community interventions for preventing prediabetes	Pre-diabetes	Knowledge about healthy lifestyles	Pasifika Prediabetes Youth Empowerment Programme: Seven empowerment modules and five co-design modules	41 Pacific youth participants in total started the programme and 29 were retained over time.	• The programme increased youth’s knowledge about health and healthy lifestyles, developed their leadership and social change capacities, and provided a tool to develop and refine culturally centred prediabetes-prevention programmes. • The programme increased the youth’s awareness about the intersectionality between mental wellness and obesity, prediabetes and Type 2 diabetes for Pasifika.
Scott 2015 [31]	Community engagement	Community members (Samoan *n* = 10, Tongan *n* = 1 & Cook Islander *n* = 1) and five Pacific health professionals.	To explore barriers to immunisation in a Pacific Island community and to conduct a pilot programme for immunisation catch-up in a Samoan church.	Immunisations	Immunisation uptake	Catch-up immunisation clinics at a Samoan church.	Attendees of a Samoan church in Western Sydney aged 7–33 years	• Approx 70 children and youth were in attendance at the first clinic held on a festival rehearsal day. There were 63 participants at the first clinic and 15 at the second. Of these, 27 at the first clinic and one at the second clinic were appropriately vaccinated; a total of 50 doses of vaccine were provided: 36 at the first clinic and 14 at the second.
Shintani 1994 [34]	Community-based philosophy Empowerment	Members of the Waianae Coast Community Committee	To describe the Waianae Diet Program, its history and current status, the theories and the practices implemented in the program that contribute to its success.	Obesity and chronic disease	Weight loss	Waianae Diet Program: Three week programme of traditional Hawaiian diet and cultural teachings.	120 individuals in Waianae, Hawaii have gone through the program since inception.	• The program demonstrated significant weight loss with no calorie restriction, improvement in blood pressure, serum glucose, and serum lipids, with wide acceptance. • After 3 weeks, the 21 participants lost an average of 17.1 pounds (*p* < 0.0001). Caloric intake decreased about 40%. Total serum cholesterol levels decreased significantly with an average decrease of 14.1%. Glucose levels reduced by 23.8% on average (*p* < 0.01). Systolic blood pressure decreased by 8.6% (*p* < 0.01) and diastolic by 10.6% (*p* < 0.01).
Simmons 2004 [35]	Community action	Two Samoan and two Tongan churches	To compare the impact on weight and exercise of a 2-year church-based diabetes risk reduction programme in four churches.	Diabetes	Weight gain	Modular lifestyle and diabetes awareness intervention.	222 members from Samoan Seventh Day Adventist 294 members from Tonga Latter Day Saints.	• In one intervention church, weight gain was controlled (vs. control 0 ± 4.8 vs.+3.1 ± 9.8 kg, respectively; *P* = 0.05), diabetes knowledge (+ 46 ± 26% vs.+4 ± 17%; *P* < 0.001) and regular exercise (at least 3 days per week: +22% vs. 8%; *P* = 0.032) increased. • The other intervention church increased diabetes knowledge (+ 19 ± 24 vs. +8 ± 25; *P* < 0.024), but no other significant changes.
Sinclair 2013 [32][	Community-based participatory research	Native Hawaiian/Pacific Islander community members and leaders	To pilot test the effectiveness of a culturally adapted diabetes self-management intervention	Diabetes self-management	A1c	Partners in Care intervention: Diabetes self-management education intervention.	82 participants (48 assigned to intervention and 34 waitlist control).	• Significant baseline-adjusted differences at 3 months between the Partners in Care intervention and waitlist control group in intent-to-treat (*p* < 0.001) and complete case analyses (*p* < 0.0001) for A1c, understanding (*p* < 0.0001), and performing diabetes self-management (*p* < 0.0001).
Tanjasiri 2019 [33]	Community-based participatory research	Leaders of Chamorro, Samoan and Tongan communities.	To test the efficacy of a unique social support intervention targeting Chamorro, Samoan, and Tongan women and their male husbands/partners.	Cervical cancer screening	Uptake of Papanicolaou (Pap) test	A single session educational intervention to increase men’s social support for their female wives/partners to receive a Pap test and for women to receive a Pap test.	Women: 591 total; 249 intervention and 342 control Men: 416 total; 150 intervention and 266 control	• Intervention women who were not compliant with Pap screening recommendations at pretest were significantly more likely to have scheduled and received a Pap test at 6-month follow-up (Beta 0.820 (SE 0.45), 95% CI −0.071, 1.171). • However, 6-month follow-up results indicated no intervention effect on changes in women’s Pap testing knowledge (*p* = 0.64), fatalistic attitudes (*p* = 0.454), or perceived social support from their male partner (*p* = 0.36).

### Co-designed health interventions

The most common health issue (*n* = 8) addressed in the interventions within this review was diabetes [21, 27–30, 32, 35, 36], with other studies covering mental health, immunisation uptake and the prevention of cancers, HIV/AIDS, malaria, food insecurity, hypertension, obesity, and other chronic diseases. All but one of the co-designed interventions were focused on or included a component of education (95%, *n* = 20), followed by physical activity [21, 24, 29, 35, 36], changing the physical environment [25, 37] and improving the provision of services [31, 38].

A variety of cultural and contextual factors were considered in the co-designed interventions. The collectivist nature of Pacific communities was commonly acknowledged alongside a strong dedication to their multigenerational families, communities, and religious beliefs [19, 24, 28, 30]. This directed interventions that were based in group-settings, incorporated peer-to-peer education [19, 24, 29, 32, 34, 38, 39], and were set in places of community importance, for example, in churches [29, 31, 33, 35], on an airstrip [37] and at community centres [33]. One medium quality randomised controlled trial involved a 12-week programme based on Hula, the traditional dance of Native Hawaiians, in order to manage patients with diagnosed hypertension [24]. The authors emphasised that hula training promoted cultural values of interconnectedness, family and spiritual beliefs, while providing a unique connection to cultural and creative expression [24]. Similarly, other studies identified respecting cultural values as paramount, with considerations given to matriarchal kinship [28], gender-related taboos [33] and talanoa (discussion) [30].

### Outcomes of participatory/co-designed interventions

A range of study designs and evaluation types were found in this scoping review, including formative evaluations, narrative evidence and self-reported structures. Overall, 19 of the 21 studies (90%) reported positive outcomes for their participatory/co-designed interventions [19–21, 23–25, 27–39].

#### Direct impacts on health

Direct changes in health status, for example weight [21, 34, 35], body fat [22, 26], glucose [27, 29, 32], blood pressure [24], mental health [23] and mortality [37], were the main outcomes measured for 11 out of 21 interventions in this review. Seven of these 11 studies had quasi/non-randomised experimental designs– five highlighting positive changes in health outcomes [21, 27, 29, 34, 35] and two reporting a lack of evidence for effectiveness [22, 26]. As an example, a high quality study by McElfish and colleagues reported a 1.45% reduction in A1C between preintervention and 12-weeks post intervention (*p* < 0.001) after evaluating a CBPR-developed video to educate Marshallese patients on blood glucose monitoring [28]. Qualitative outcomes also showed that participants found the educational approach to be informative, and all agreed that it was culturally appropriate and effective [28]. Two medium quality randomised controlled trials with the primary outcomes of blood pressure [24] and HbA1c [32] also reported positive outcomes. Sinclair and colleagues aimed to improve knowledge, understanding and skills through a culturally relevant programme to support Native Hawaiian and Pacific people’s self-management of diabetes [32]. Although the effect size was noted to be small, the authors found a significant difference in A1C between the intervention and control group after three months [32]. Other study designs included a qualitative study on mental health outcomes [23] and a narrative case study focused on malaria-related deaths, both of which reported improvements as a result of the participatory/co-designed interventions [37]. In general, the 11 studies measuring direct health outcomes were of high quality (*n* = 7), with three medium quality and one lower quality.

#### Indirect health outcomes

Ten studies of the 21 in this review measured indirect impacts on health as their main outcome. These included screening or immunisation uptake [19, 31, 33, 39], improvement in attitudes, self-efficacy and awareness [28, 30, 38] and changes in diet/physical activity [20, 25, 36] and all ten interventions reported positive outcomes. Four were quasi/non-randomised experimental studies [19, 20, 25, 28], with two each of high and medium quality. An example of a high quality study by Aiaoto and colleagues showed how CBPR was used to develop training and education materials to encourage the uptake of mammograms for Micronesian populations [19]. Screening compliance increased in women who were eligible for mammogram screening (i.e. those aged 40 years or older, *n* = 202), from a baseline of 18–90% after six months of the programme [19]. Two randomised controlled trials measured utilisation of pap tests (one high quality and one medium quality) [33, 39]. The high quality trial used CBPR and empowerment models to develop cervical cancer educational approaches in American Samoa [39]. Those women receiving education in the intervention group had two times higher odds of obtaining a pap smear six months after the intervention compared to those in the control group (*p* < 0.01) [39]. Other study designs measuring indirect impacts on health included three qualitative studies of mixed quality [30, 31, 36] and one high quality case report study [38].

#### Common limitations

While most studies in this review reported positive health outcomes resulting from co-designed interventions, common limitations were identified that restricted the applicability and generalisability of the findings. Seven studies acknowledged that short timeframes, due to funding, research, or cultural factors, limited the ability for extended observations, intervention implementation and follow-up [19, 21, 23, 25, 26, 29, 36]. Additionally, seven studies acknowledged the potential for small sample sizes to impact study findings [20, 21, 23, 27–29, 31]. For example, Ndwiga and colleagues evaluated a church-based diabetes education and support programme for Australian Samoans living in Sydney [29]. Although the study showed a significant drop of HbA1c from baseline to follow-up among participants with diabetes, no significant reductions were found for blood pressure, body mass index or waist circumference. The authors concluded that larger, more long term studies were needed to maximise the effectiveness [29]. The two studies in this review that reported a lack of effectiveness for their interventions were quasi/non-randomised experimental designs [22, 26], with both citing the need for more comprehensive approaches that account for sociocultural factors and cultural expectations. Kremer and colleagues stated, “the strength of the intervention may have been insufficient to combat the prevailing physical, economic and sociocultural forces that contribute to unhealthy weight gain among Fijian adolescents” [26, p. 38]].

## Discussion

This scoping review aimed to map the scope of existing studies on health interventions co-designed by Pacific communities globally. Twenty-one studies met the inclusion criteria for this review, with interventions conducted across nine different countries and ten different Pacific ethnicities participating in the development process. Final studies in this review primarily focused on Pacific populations in high-income, Western countries and most had quasi-experimental study designs. Such characteristics align with existing literature, for example a systematic review on behaviour change interventions which found that most Pacific research occurred in the United States and had non-randomised designs [40]. This highlights a need for research in low- and middle-income countries with more rigorous study designs to strengthen the evidence base of interventions.

A range of participatory approaches to co-designing interventions were identified, with CBPR being the most used with Pacific communities. Of the 13 studies that provided definitions for the methodology used, most outlined an equal partnership model that aimed to engage communities throughout the entire research journey, build capacity and empower mobilisation. Descriptions of the Pacific community groups involved, and the process of co-developing interventions were not well reported across the studies, with very few including information on when the partnership began, who initiated it and the frequency of meetings. Slattery and colleagues identified similar findings in their review of co-designed health research, stating that vague terminology and insufficient descriptions of co-design processes made it difficult to understand what procedures were involved [3]. The lack of a standardised process also made it difficult for other researchers to incorporate these activities into their own research [3]. Including definitions and more standardised descriptions of the processes undertaken with Pacific communities would enable more clarity around the engagement process and direction for future research. Developing a clear, shared international definition of co-design and other participatory processes, alongside a set of high-level principles that describes what good practice looks like is necessary to address the heterogeneity in the field. Further to this, detailed descriptions of the process, providing the rationale for decisions and changes, and openly discussing how community engagement and relationship building were undertaken is vital to supporting the meaningful evaluation of co-design.

Pacific cultural factors were considered in how participatory methods were conducted, such as discussion-based methodologies reflecting oral traditions, separating groups by gender, and incorporating prayer, singing and food. This was similarly reflected in the composition of co-designed interventions in this review, which, in addition to these factors, prioritised cultural values and traditions, were mindful of taboos, and considered settings of community importance for intervention implementation. A study by Suarez-Balcazar and colleagues similarly recognised the importance of cultural tailoring in the implementation of a health promotion programme for Latino youth and their families [41]. The authors discussed that while traditional behaviour management approaches are often rooted in the Western values of independence and self-control, Mexican families value collectivism and familism, and interventions should reflect this [41]. Participatory research frameworks such as the approaches described in this review, provide an important opportunity for interventions to not only be led by the community it aims to benefit, but also to ensure they are culturally relevant, respectful, and ultimately have the potential to be impactful. Researchers undertaking co-design methods with Pacific or other minority communities should ensure processes incorporate cultural factors in intervention development and implementation and equally evaluate these factors to better inform understandings of how cultural tailoring supports improved health outcomes.

Overall, most interventions outlined in the review papers reported positive impacts on health, highlighting the potential of participatory processes to benefit researchers, build capacity amongst Pacific people, and importantly positively affect the health of the wider Pacific community. Studies discussed the benefits of community leadership and champions for the success of interventions. This peer-to-peer education was critical, as the existing strong relationships between community leaders and members enabled ownership and trust of the programme. Historically, the voices of Indigenous and other minority communities have been excluded from traditional research processes, with experiences of limited control over the portrayal and uses of community data, ultimately fuelling a lack of trust in research [42]. Ensuring that researchers take time to build strong relationships and empower participation of Pacific communities from an early stage is crucial to achieving lasting partnerships. More high-quality research in this area would be beneficial to inform guidance and protocols for evaluating co-designed processes, thus enabling better understandings of which approaches are most effective and why.

### Strengths and limitations

A strength of this scoping review is the broad scope that enabled mapping of different Pacific co-design approaches undertaken across a range of health issues. This allowed key definitions and processes to be examined, exploring the similarities and differences of implementation. Although the positive outcomes indicate the success of participatory approaches and prioritising community knowledge, it is important to acknowledge the range of information sources included and the evaluative structures used in studies, their limitations and the overall quality. Although grey literature was included, the search is limited to types of evidence that is typically accessible online, such as websites, governmental reports and theses repositories. Evidence that is reported internally in organisations, or that may be in hard copy form only would therefore be missed from this review. The success of the interventions in this study may also be linked to publication bias whereby non-significant results may be less likely to be published or reported on organisation websites.

Key principles in CBPR, participatory action research and co-design overlap with traditional Pacific values (for example collective decision-making and reciprocal relationships), however it is important to acknowledge that these methodologies are inherently Western frameworks. While one study in this review acknowledged the inclusion of a Pacific framework in its programme, the Fonofale model [30], a limitation of this review is the potential for relevant studies to be missed if they focus solely on Pacific methodologies and do not specify the Western participatory terms included in this search strategy. Differing definitions of “Pacific” is also important to note, due to definitions primarily in the United States that often combine Asian Americans and Pacific Islanders and thus were not able to be included.

### Implications

This review has shown that clearer definitions, descriptions, and reporting of participatory processes with Pacific communities are urgently needed for studies moving forward. When co-designing with and for collective cultures, such as Pacific, efforts must be made to draw on the participatory and shared knowledge creation practices and epistemological frameworks already being operationalised within these communities. Having said that, care must be taken not to co-opt these epistemological frameworks and shared approaches; an orientation toward co-design with and for Pacific communities, must, by necessity, position Pacific communities as experts. This review found a disproportionate number of studies conducted in high income countries versus those in lower income countries, highlighting a need for more research in these settings. To accurately capture and report on the effectiveness of resulting interventions, more rigorous study designs, such as randomised controlled trials should be prioritised alongside comprehensive evaluation structures embedded from the beginning of the projects. Larger sample sizes, longer timeframes for observation and gold standard methods are critical to improve the efficacy of Pacific health co-design projects and will provide clearer direction for future research projects. Although the processes outlined in this review represent community participatory approaches, many are still initiated by researchers and governmental agencies based on their own priorities. Organisations and funding bodies should strive to enable communities the freedom to consider what health issues are most important to them and in turn, support the development of community interventions from these ideas that can be evaluated over a long time period.

## Conclusions

This review has mapped existing evidence regarding co-design health interventions undertaken with and for Pacific communities. The studies included in this review addressed a range of health issues affecting Pacific people, such as diabetes management, hypertension, and cancer screening, and by and large were successful in achieving positive outcomes. Moving forward, researchers should prioritise clearer definitions and descriptions of the participatory process and incorporate high quality evaluation plans from the outset. This review has shown that when Pacific communities are enabled and empowered to create solutions for their own communities, positive shifts in health can occur.

## Supplementary Information

Additional file 1. Scoping review checklist

Additional file 2. Search strategy

Additional file 3. Quality assessment

Additional file 4. Data extraction tables

## Data Availability

All data generated or analysed during this study are included in this article [and its supplementary information files].
