# Supplementary material for: The processes and impacts of co-designed health interventions by and for Pacific populations: a scoping review
Source: BMC Public Health. 2025 Jul 26;25:2555. doi: 10.1186/s12889-025-23795-w (PMC12297805; doi:10.1186/s12889-025-23795-w)
Supplement: Supplementary file 2 — Additional file 2. Search strategy [file 12889_2025_23795_MOESM2_ESM.docx]

## Additional files 3: Quality assessment

**Randomised controlled trials**

| **JBI Checklist** | **Mishra 2009** | **Tanjasiri 2019** | **Kaholokula 2017** | **Sinclair 2013** |
| --- | --- | --- | --- | --- |
| 1. Was true randomization used for assignment of participants to treatment groups? | Unclear | Yes | Yes | Yes |
| 1. Was allocation to treatment groups concealed? | Unclear | No | Unclear | Unclear |
| 1. Were treatment groups similar at the baseline? | Yes | Yes | Yes | Yes |
| 1. Were participants blind to treatment assignment? | Yes | No | Unclear | Unclear |
| 1. Were those delivering treatment blind to treatment assignment? | Unclear | No | Unclear | Unclear |
| 1. Were outcomes assessors blind to treatment assignment? | Yes | No | Unclear | Unclear |
| 1. Were treatment groups treated identically other than the intervention of interest? | Yes | Yes | Yes | Yes |
| 1. Was follow up complete and if not, were differences between groups in terms of their follow up adequately described and analysed? | Yes | Yes | Yes | Yes |
| 1. Were participants analysed in the groups to which they were randomized? | Yes | Yes | Yes | Yes |
| 10.Were outcomes measured in the same way for treatment groups? | Yes | Yes | Yes | Yes |
| 11.Were outcomes measured in a reliable way? | Yes | Unclear | Yes | Yes |
| 12.Was appropriate statistical analysis used | Yes | Yes | Yes | Unclear |
| 13.Was the trial design appropriate, and any deviations from the standard RCT design (individual randomization, parallel groups) accounted for in the conduct and analysis of the trial? | Yes | Yes | Yes | Yes |
| Overall Appraisal | 10/13 | 8/13 | 9/13 | 8/13 |
|  | High | Medium | Medium | Medium |

**Quasi/non-randomised experimental studies**

| **JBI Check list** | **Shintani 1994** | **Simmons 2003** | **Kaufer 2010** | **Fotu 2011** | **Kremer 2011** | **Aitaoto 2012** | **McElfish 2015** | **McElfish 2019** | **Ndwiga 2020** | **Firestone 2022** | **Chung-Do 2024** |
| --- | --- | --- | --- | --- | --- | --- | --- | --- | --- | --- | --- |
| 1. Is it clear in the study what is the ‘cause’ and what is the ‘effect’ (i.e. there is no confusion about which variable comes first)? | Yes | Yes | Yes | Yes | Yes | Yes | Yes | Yes | Yes | Yes | Yes |
| 1. Were the participants included in any comparisons similar? | Unclear | Yes | Yes | Yes | Yes | Yes | Yes | Yes | Yes | Yes | Yes |
| 1. Were the participants included in any comparisons receiving similar treatment/ care, other than the exposure or intervention of interest? | Unclear | Yes | Unclear | Yes | Yes | Yes | Yes | Yes | Yes | Yes | Unclear |
| 1. Was there a control group? | No | Yes | No | Yes | Yes | No | No | No | No | No | No |
| 1. Were there multiple measurements of the outcome both pre and post the intervention /exposure? | Yes | Yes | Yes | Yes | Yes | Unclear | Yes | Yes | Yes | Yes | Yes |
| 1. Was follow up complete and if not, were differences between groups in terms of their follow up adequately described and analyzed? | Unclear | Yes | Unclear | Yes | Yes | Yes | Yes | Yes | Yes | Unclear | Yes |
| 1. Were the outcomes of participants included in any comparisons measured in the same way? | Yes | Yes | Yes | Yes | Yes | Yes | Yes | Yes | Yes | Yes | Yes |
| 1. Were outcomes measured in a reliable way? | Unclear | Yes | Unclear | Yes | Yes | Yes | Yes | Yes | Yes | Yes | No |
| 1. Was appropriate statistical analysis used? | No | Unclear | Yes | Yes | Yes | Yes | Yes | Yes | Yes | Yes | No |
| Overall Appraisal | 3/9 | 8/9 | 5/9 | 9/9 | 9/9 | 7/9 | 8/9 | 8/9 | 8/9 | 7/9 | 5/9 |
|  | Low | High | Medium | High | High | High | High | High | High | High | Medium |

**Qualitative research design**

| **JBI Checklist** | **Oliver 2007** | **Prapaveissis 2022** | **Scott 2015** | **Han 2015** |
| --- | --- | --- | --- | --- |
| 1. Is there congruity between the stated philosophical perspective and the research methodology? | Unclear | Unclear | Unclear | Unclear |
| 1. Is there congruity between the research methodology and the research question or objectives? | Yes | Yes | Unclear | Yes |
| 1. Is there congruity between the research methodology and the methods used to collect data? | Yes | Yes | Unclear | Yes |
| 1. Is there congruity between the research methodology and the representation and analysis of data? | Yes | Yes | Unclear | Yes |
| 1. Is there congruity between the research methodology and the interpretation of results? | Yes | Yes | Unclear | Yes |
| 1. Is there a statement locating the researcher culturally or theoretically? | No | Yes | No | No |
| 1. Is the influence of the researcher on the research, and vice- versa, addressed? | No | No | No | No |
| 1. Are participants, and their voices, adequately represented? | Yes | Yes | Yes | No |
| 1. Is the research ethical according to current criteria or, for recent studies, and is there evidence of ethical approval by an appropriate body? | No | No | Yes | No |
| 1. Do the conclusions drawn in the research report flow from the analysis, or interpretation, of the data? | Yes | Yes | Yes | Yes |
| Overall Appraisal | 6/10 | 7/10 | 3/10 | 5/10 |
|  | Medium | High | Low | Medium |

**Textual evidence: Narrative**

| **JBI Checklist** | **Fitzpatrick 2007** | **Katz 2007** |
| --- | --- | --- |
| 1. Is the generator of the narrative a credible or appropriate source? | Yes | Yes |
| 1. Is the relationship between the text and its context explained? (where, when, who with, how) | Yes | Yes |
| 1. Does the narrative present the events using a logical sequence so the reader or listener can understand how it unfolds? | Yes | Yes |
| 1. Do you, as reader or listener of the narrative, arrive at similar conclusions to those drawn by the narrator? | Yes | Yes |
| 1. Do the conclusions flow from the narrative account? | Yes | Yes |
| 1. Do you consider this account to be a narrative? | Yes | Yes |
| Overall Appraisal | 6/6 | 6/6 |
| Comments | High | High |
