# Supplementary material for: The processes and impacts of co-designed health interventions by and for Pacific populations: a scoping review
Source: BMC Public Health. 2025 Jul 26;25:2555. doi: 10.1186/s12889-025-23795-w (PMC12297805; doi:10.1186/s12889-025-23795-w)
Supplement: Supplementary file 3 — Additional file 3. Quality assessment [file 12889_2025_23795_MOESM3_ESM.docx]

## **Additional file 4: Data extraction tables**

Additional files table 1: Characteristics information for included studies

| **Author/Year** | **Title** | **Year published** | **Country** | **Village/town/city/state** | **Aim of study** | **Study design** |
| --- | --- | --- | --- | --- | --- | --- |
| Aitaoto 2012 | Design and results of a culturally tailored cancer outreach project by and for Micronesian women | 2012 | United States | Hawai'i | To develop and track the reach of Micronesian women lay educators in implementing a cancer awareness program among Micronesian women living in Hawaii. | Non-randomised experimental study design |
| Chung-Do 2024 | MALAMA: Cultivating Food Sovereignty through Backyard Aquaponics with Native Hawaiian Families | 2024 | United States | Hawai’i | To test the feasibility and acceptability of the MALAMA program as a public health intervention to promote healthy eating. | Pre-post study design |
| Firestone 2021 | Pasifika Prediabetes Youth Empowerment Programme: learnings from a youth-led community-based intervention study | 2021 | New Zealand | Auckland and South Waikato | To co-design a small-scale community-based intervention, led by the Pasifika youth and implement and evaluate the short-term success of the interventions. | Pre-post interventional study |
| Fitzpatrick 2007 | Empowering the initiation of a prevention strategy to combat malaria in Papua New Guinea | 2007 | Papua New Guinea | Batri village | To explore how an empowerment approach could affect changes in malaria prevalence among remote members of the Kewapi language group in PNG. | Project report/Case study |
| Fotu 2011 | Outcome results for the Ma'alahi Youth Project, a Tongan community-based obesity prevention programme for adolescents | 2011 | Tonga | Tongatapu and Vava'u | To present the results of the Ma'alahi Youth Project, the first community-based intervention to target adolescent obesity in the Kingdom of Tonga. | Quasi-experimental design with a longitudinal cohort follow-up |
| Han 2015 | An innovative community organizing campaign to improve mental health and wellbeing among Pacific Island youth in South Auckland, New Zealand | 2015 | New Zealand | South Auckland | To improve the mental health outcomes of low-income minority youth by engaging them in community organizing. | Pre-post study design |
| Kaholokula 2017 | Cultural Dance Program Improves Hypertension Management for Native Hawaiians and Pacific Islanders: a Pilot Randomized Trial | 2017 | United States | Honolulu, Hawai'i | To test both feasibility and efficacy of Ola Hou in reducing the blood pressure in a community sample of Native Hawaiian Pacific Islanders with physician-diagnosed hypertension. | Randomised controlled trial |
| Katz 2007 | Putting the community at the center of measuring change in HIV prevention in Papua New Guinea: the Tingim Laip (Think of Life) Mobilisation | 2007 | Papua New Guinea | Country-wide | To describe the Tingim Laip intervention,  the participatory monitoring and evaluation mechanisms, and some of the preliminary findings. | Case report |
| Kaufer 2010 | Evaluation of a "traditional food for health" intervention in Pohnpei, Federated States of Micronesia | 2010 | Federated States of Micronesia | Pohnpei | To assess changes in diet and health that may have been impacted by the two-year intervention. | Pre-post study design |
| Kremer 2011 | Reducing unhealthy weight gain in Fijian adolescents: Results of the Healthy Youth Healthy Communities study | 2011 | Fiji | Sigatoka, Nadi and Lautoka | To report on the outcomes of a 3-year obesity prevention study, Healthy Youth Healthy Communities undertaken with Fijian adolescents | Non-randomised experimental study |
| McElfish 2015 | Family model of diabetes education with a Pacific Islander community | 2015 | United States | Arkansas | To use a community-based participatory approach to pilot test a family model of diabetes education conducted in participants homes with extended family members. | Pre-post study design |
| McElfish 2019 | Development and Evaluation of a Blood Glucose Monitoring YouTube Video for Marshallese Patients Using a Community-Based Participatory Research Approach | 2019 | United States | Arkansas | To develop and test a health education  video focused on blood glucose monitoring and  control. | Pre-post study design |
| Mishra 2009 | Increasing pap smear utilization among Samoan women: Results from a community based participatory randomized trial | 2009 | American Samoa | Eastern and Western Districts of the main island of Tutuila | To test the effectiveness of a theory-guided, culturally tailored cervical cancer education program designed to increase Pap smear use. | Randomised controlled trial |
| Ndwiga 2020 | Outcomes of a church-based lifestyle intervention among Australian Samoans in Sydney: Le Taeao Afua diabetes prevention program | 2020 | Australia | Sydney | To evaluate the effectiveness of a culturally adapted, church-based lifestyle intervention among Australian Samoans living in Sydney. | Prospective pre-post study |
| Oliver 2007 | Stories from the past, the reality of the present, taking control of the future' - Lifestyle changes among Pukapuka people in the Illawarra | 2007 | Australia | Illawarra region, New South Wales | To develop appropriate interventions to increase physical activity rates and knowledge of nutrition. | Qualitative research design |
| Prapaveissis 2022 | Assessing youth empowerment and co-design to advance Pasifika health: a qualitative research study in New Zealand | 2022 | New Zealand | Tokoroa and Henderson, Auckland | To investigate empowerment and co-design modules to build the capacity of Pasifika youth to develop community interventions for preventing prediabetes | Qualitative research design |
| Scott 2015 | Responding to a measles outbreak in a Pacific Island community in western Sydney: community interviews led to church-based immunization clinics | 2015 | Australia | Western Sydney | To explore barriers to immunisation in a Pacific Island community and to conduct a pilot programme for immunisation catch-up in a Samoan church. | Qualitative research design |
| Shintani 1994 | The Waianae Diet Program: a culturally sensitive, community-based obesity and clinical intervention program for the Native Hawaiian population | 1994 | United States | Hawai'i | To describe the Waianae Diet Program, its history and current status, the theories and the practices implemented in the program that contribute to its success. | Pre-post intervention study |
| Simmons 2004 | Tale of two churches: differential impact of a church-based diabetes control programme among Pacific Islands people in New Zealand | 2004 | New Zealand | South Auckland | To compare the impact on weight and exercise of a 2-year church-based diabetes risk reduction programme in four churches. | Prospective non-randomised controlled study |
| Sinclair 2013 | Outcomes from a diabetes self-management intervention for native Hawaiians and pacific people: Partners in care | 2013 | United States | Hawaii | To pilot test the effectiveness of a culturally adapted diabetes self-management intervention | Randomised controlled trial |
| Tanjasiri 2019 | Design and outcomes of a community trial to increase pap testing in Pacific Islander women | 2019 | United States | California | To test the efficacy of a unique social support intervention targeting Chamorro, Samoan, and Tongan women and their male husbands/partners. | Randomised controlled trial |

Additional files table 2: Information on participatory processes

| **Author/Year** | **Start of partnership** | **Who initiated the partnership process?** | **Types of stakeholders involved** | **Pacific co-design partners** | **Nature of co-design process** | **Participatory method(s) stated** | **Definition of method(s)** | **Cultural considerations in CBPR/co-design process** |
| --- | --- | --- | --- | --- | --- | --- | --- | --- |
| Aitaoto 2012 | 2005 | Micronesians United, a grassroots organisation, and Imi Hale Native Hawaiian Cancer Network. Micronesians United asked Imi Hale for help to increase breast cancer awareness and screening. | Community members  Community organisations/service providers | 16 Micronesian women (aged 22-69). | A focus group with 16 women in October 2005 to identify leading health concerns, barriers to good health and cultural strengths that can improve the health of Micronesians in Hawaii. Breast cancer was identified by 15 of the 16 women.  'Champions' reviewed literature on lay educator cancer programs and considered tailoring and translations for relevance to Micronesian women. The finalised toolkit materials were tested with 80 Micronesian women. | Community-based participatory research | Guided by principles of community-based participatory research, emphasising community involvement, capacity building, respect for cultural values, and information sharing.  Adhering to community-based participatory research principles, the development process included four steps: 1) building relationships and identifying champions; 2) working together to assess needs, barriers, and desired solutions; 3) culturally tailoring materials and tools; and 4) training lay educators. | Using methods that worked previously in the Samoan community in Hawai‘i, each focus group session was started with a prayer, served food, and allowed the group to determine the amount of time spent on each question. The focus group was conducted in English, with assistance of translators for Chuukese, Marshallese, Pohnpeians and Kosraean. |
| Chung-Do 2024 | 2017 | Ke Kula Nui O Waimānalo | Community members  Community organisation  Researchers & academics  University students | Community leaders and residents of Waimānalo | Ke Kula Nui O Waimānalo is a grassroots nonprofit organization that was founded in 2017 by community leaders and partners of Waimānalo.  KKNOW also houses the Waimānalo Pono Research Hui which is a community–academic partnership that promotes community-driven and culturally-grounded research | Community-based participatory research | No definition or explanation provided. | None stated |
| Firestone 2021 | Unknown | Research team | Researchers & academics  Health providers  Community members  Youth | 41 young Pasifika youth (15-24 years) from an Auckland health provider and a rural Waikato health provider | Pasifika Prediabetes Youth Empowerment Programme: the group of 41 Pasifika youth co-designed action plans to reduce prediabetes risk factors in their communities. The youth participated for 2–2.5 hours per week throughout the five-month empowerment programme, where they developed practical skills and knowledge through the modules. In the second phase, the youth translated these action plans into community-based intervention programmes and delivered them in their communities. | Community-based partnership  Participatory action research  Empowerment  Co-design | - Community-based partnerships are essential to address inequities and to explore culturally appropriate services that are community-based. - Empowering Pacific communities to participate in all stages of any proposed research will enhance intervention development, engagement and uptake and provide evidence-based knowledge. - The research approach uses an established empowerment framework that was uniquely designed to build the health-leadership capacity of Pasifika youth, transform their knowledge and skillsets into actionable knowledge and ultimately mobilise their communities towards a common purpose. | Prior to the project, four Pasifika facilitators were trained extensively to upskill their expertise on how to engage with Pasifika youth, facilitate discussions and deliver the piloted empowerment modular programme. |
| Fitzpatrick 2007 | 2004 | Urban members of the Kewapi language group | Researchers  Community members  Government organisations | Members of the Kewapi language group living in urban Erima, Port Moresby | The genesis of the project was the concern of members of the Kewapi language group living in urban Erima, Port Moresby, for those remaining in their home village, Batri, suffering the effects of malaria. There was concern that those in Batri had to wait several years to acquire the bed nets provided by the Global Fund to Fight AIDS, Tuberculosis and Malaria. The Erima group became part of an 'empowerment research project' in 2004-2005: the Erima Empowerment Research Project. A collaborative research approach by members of the initial Erima Empowerment Research Project led to the 'Batri Village Bed Net Initiative'. | Empowerment | Empowerment research enables members of communities to take ownership of a project agenda and develop relevant initiatives. In this way, disadvantaged communities feel that their concerns are heard, enabling them to engage with political agendas that affect community development and bring about change. | Elders from the language group fluent in English Pidgin an Kewapi and familiar with the cultural norms of the village, including the tribal discussion system, enabled project members to communicate effectively.  Strengths included capitalising on the traditional discussion forum of the community, leading to a supportive culture that enabled mutual sharing and exploration of issues. |
| Fotu 2011 | 2005 | Research team | Researchers and academics  Community members   Government organisations or NGOs   Schools  Youth | Tongan community members, youth leaders, ministers, parents, town officers and health representatives. | The Ma’alahi Youth Project aimed to build the capacity of communities and schools to create their own solutions. While the MYP objectives were common to the three Tongan intervention sites, the implementation processes were contextualized for each school and community.  Further details published elsewhere. | Community capacity building | The Ma’alahi Youth Project aimed to build the capacity of communities and schools to create their own solutions for promoting healthy eating, physical activity and healthy weight gain in adolescents aged 11-19 years and their families | None stated |
| Han 2015 | 2012 | A health provider, Counties Manukau Health | Researchers and academics  Health providers  Youth | A Pacific youth as a full-time lead organiser and a leadership team of five Pacific youths. | Counties Manukau Health (CM Health) hired a Pacific youth as a full-time lead organiser in August 2012, with coaching and support from the United States. The lead organiser recruited a team of five volunteer youths to form a leadership team, and this team designed the campaign.  The youth leaders thus developed a campaign called Handle the Jandal. The first job of the five youth leaders was for each of them to recruit five other youths, and train them in the principles and practices of organising. The leadership team worked to recruit 25 youths to the campaign. 21 of the 25 recruits and the five-member leadership team participated in two days of training. | Community organising | - Unlike other approaches to social change, community organising makes change possible by developing the capacity of a constituency to act on their own health – in other words, by developing their individual and collective agency. - Traditional approaches try to create change without developing agency within the constituency they are seeking to help. Community organising is distinct from other approaches to change because it is built on the assumption that long-term change occurs by developing the agency of the constituency who wants the change. In the context of health, community organising can help develop the agency individuals and communities have over their own health, thus improving health outcomes. | None stated. |
| Kaholokula 2017 | Unknown | Academic and community researchers in the Hula Empowering Lifestyle Adaptations Project | Researchers and academics  Community members | A kumu hula (hula expert) and two Native Hawaiian Pacific Islander community leaders | A community-based participatory research approach was used in designing and implementing this study. Community investigators included a kumu hula (hula expert) and two NHPI community leaders, while our academic investigators included a cardiologist, psychologist, and public health researchers. | Community-based participatory research | No definition or explanation provided. | None stated. |
| Katz 2007 | 2005 | A government organisation, the National AIDS Council | Community members  Community organisations/service providers  Government organisations  Youth | Papua New Guinean (PNG) "change agents" and local communities | This project is the result of a partnership between the National AIDS Council PNG, National HIV/AIDS Support Project, Australian Agency for International Development, Family Health International and hundreds of "change agents" in the network of Tingim Laip sites throughout Papua New Guinea. | Action-oriented  Participatory action  Community mobilisation  Capacity building  Bottom-up approach | - Action oriented communication includes interpersonal communication and peer education, participatory learning and action, advocacy and community mobilization. - A bottom-up approach was taken to communication priorities, strategies and messages. Local communities develop messages according to local needs, realities, interests, customs, and languages with technical support provided as required. This approach helps to ensure that local meaning is given to messages helping to support attitudinal, behavioural and social change. - The mobilisation focuses on building capacity of those spearheading the intervention (change agents) in each of the settings. - Emphasis of the participatory monitoring and evaluation is on empowering the community settings to decide how they wanted to measure change by providing those who are spearheading interventions with a tool-kit of methodologies and letting them decide what is most useful to their own interventions, capacity, and needs. This flexible and participatory approach motivated communities to drive the process. | None stated. |
| Kaufer 2010 | Unknown | Pohnpei agencies and the Centre for Indigenous Peoples' Nutrition and Environment at McGill University, Canada. | Researchers and academics  Community members  Community organisations/service providers  Government organisations  Health providers | Mand community leaders and residents | The Island Food Community of Pohnpei and other Pohnpei agencies worked with the Mand community on the island of Pohnpei and the Centre for Indigenous Peoples' Nutrition and Environment's Indigenous Peoples' Food Systems for Health program.   A participatory, food-based approach was used to assist the residents of Mand to meet their food needs, increase self-reliance, improve health and nutrition, economic savings and protect agricultural biodiversity. | Participatory approach | No definition or explanation provided. | None stated. |
| Kremer 2011 | 2004 | Research team | Researchers and academics  Schools and students  Community members  Churches | Fijian schools, students, teachers, community members, local council representative | During August 2004, a workshop was conducted in the wider community and the subsequent action plan comprised behavioural objectives where children were encouraged on a variety of healthy behaviours e.g. diet, TV watching and active play.   The intervention programs in communities were developed in collaboration with the wider community, primarily through the faith-based organisations. The community intervention research assistants worked through the umbrella group of different religious groups (religious leaders) to gain access to women's groups and youth groups affiliated with each denomination. | Community capacity building | Community capacity building refers to the development of knowledge, skills, commitment, structures, systems, and leadership to enable effective health promotion which may enhance the ability of a community to establish and deliver a programme, to maintain and sustain a programme, or to problem-solve and develop programmes for other health issues. | None stated. |
| McElfish 2015 | 2012 | The research team (University of Arkansas for Medical Sciences Northwest) | Researchers/academics/universities  Community members  Health providers | Marshallese community  A Marshallese community leader and health worker on study team. | In 2012, the University began engaging the Arkansas Marshallese in a community-based participatory research process to understand what health disparities the community wanted to address. The Marshallese stakeholders chose diabetes.   The interprofessional community-based participatory research study team was comprised of five clinical faculty (one nurse/certified diabetes educator, two pharmacists, and two endocrinologists), two Marshallese community co-investigators (one community leader and one community health worker), and the lead investigator of the community-based participatory research partnership. | Community-based participatory research | No definition or explanation provided. | None stated. |
| McElfish 2019 | 2013 | The research team (University of Arkansas for Medical Sciences) | Researchers/academics/universities  Community members  Health providers | 9 Marshallese community members and health professionals | The lead author collaboratively developed the video script and format with five Marshallese community members, two Marshallese community health workers, a Marshallese family practice physician, and a Pacific Islander health education researcher. | Community-based participatory research | Community-based participatory research engages nontraditional partners and honours their unique contributions at all phases of the research process from prioritizing the research needs to disseminating the findings.  Community-based participatory research has demonstrated effectiveness in building alliances with minority, immigrant communities to improve health when there are disparities resulting from systematic disadvantage, racism, and historical trauma. | Community-based participatory research has demonstrated effectiveness in building alliances with minority, immigrant communities to improve health when there are disparities resulting from systematic disadvantage, racism, and historical trauma. |
| Mishra 2009 | Unknown | The National Office of Samoan Affairs (a community-based service organization based in Los Angeles, community partner) and the University of California-Irvine (academic partner) participated in this community-academic collaborative effort.  Both groups had previously collaborated on research for seven years | Researchers/academics/universities  Community members  Community organisations/service providers | Samoan women | The intervention was a result of community-identified needs.   The project constituted a Community Advisory Committee which provided leadership and guidance to the project and was chaired by a community member.  The project's "community" comprised Samoan women. They participated in the project as co- Principal Investigator (co-PI), chair of the Community Advisory Committee, members of the Community Advisory Committee, cultural and linguistic experts, translators, recruiters of potential study sites, recruiters of eligible women for the study, interviewers, health educators, trainers, data managers, and administrators. | Community-based participatory research  Empowerment | - No definition provided for community-based participatory research. - The Freirian empowerment pedagogy provides a model for adult education and it defines attributes of an ideal education environment, the mode of effective education and nurturance of new behavioural skills, and the incorporation of social and cultural norms and beliefs within the educational context. The pedagogy is based on the problem-posing learning method and involves an interactive rather than a didactic model of learning. There is extensive open dialogue, active participation, and group involvement, which allows the learners to become more personally involved in the subject of interest and yielding an increased likelihood of application of the new knowledge in everyday life. In this pedagogy, learners are encouraged to appraise critically and to internalize new information they receive. | The empowerment pedagogy used in the community-based participatory process was appropriate for Samoans since they have an oral tradition that values collective decision-making, experiential education, trust building, and interpersonal interactions. |
| Ndwiga 2020 | Unknown | The research team | Researchers/academics/universities  Community members | Samoan community representative reference group | A Samoan community representative reference group met every 3-4 months to guide this project. | Community-based participatory research  Co-design | The study adopted the community-based participatory framework which facilitates an active co-design approach allowing researchers and stakeholders to engage and adapt practices to suit community needs and empowers community members to take ownership of the project. | The study adopted the community-based participatory framework which has been used successfully in other Pacific intervention studies. |
| Oliver 2007 | Unknown | The research team | Researchers/academics/universities  Community members | 24 Cook Islanders in the Illawarra region (13 women and 11 men) | Participants were recruited using a convenience sample of Cook Islanders in the Illawarra. Thirteen women and 11 men participated in a study over an eight-month period, including an eight-week intervention phase. The design stage involved a series of focus group discussions that allowed individuals to tell their own stories and have them validated by the group. The first groups were used to develop a common understanding of how tradition, westernisation and migration have affected the lifestyle of Pukapuka people.   The outcomes from the initial discussions were analysed and fed back to participants during subsequent sessions for analysis and reflection. These results were used to jointly plan activities that respected cultural and socio-economic factors. | Action research | This study followed Stringer and Dwyer’s Action Research framework. The five steps – designing, collecting data, analysing data, communicating outcomes and taking action are completed in an iterative and collaborative process to incorporate the views, perspectives and experiences of clients to resolve a community problem. | In addition to discussions with the research team, leaders were employed as focus group facilitators to ensure that cultural expectations were respected. Community leaders indicated that separate male and female groups were necessary to permit open discussion.  Focus groups started with a light dinner as a way of demonstrating respect and friendship. Traditions such as prayer and song were observed to open and close the sessions. The groups became the first step towards the development of trust between researchers and the community. |
| Prapaveissis 2022 | Unknown | The research team (Massey University) and two Pasifika community health service providers. | Researchers/academics/universities  Community members  Health providers  Youth | Pacific health providers and community partners | The Pasifika Prediabetes Youth Empowerment Programme is a partnership between researchers at Massey University and two Pasifika community health service providers, one in an urban and the other in a rural location in New Zealand. The community service organisations herein referred to as the 'community partners' hired a community research facilitator who led the project with three research assistants from Massey University and guidance from the principal investigators. The community partners led the recruitment and engagement strategy, utilising their existing relationships with schools, churches and youth organisations to recruit participants. | Community-based participatory research  Co-design  Empowerment | - Empowerment-based interventions, defined as interventions aimed to build capacity, redistribute power, and educate people, consistently demonstrate promise in health promotion among peoples that experience marginalisation. They take a health-enhancing approach and often address broader determinants of health and have been described as a mechanism for social change. - Co-design is an innovative, interdisciplinary approach to develop, test, and implement innovative systems, programmes, tools or products. It takes a bottom-up approach to collaboratively develop initiatives with stakeholders that would have been traditionally underrepresented or not included in designing and implementing the intervention. Co-design has demonstrated success with young people to initiate community change, as well as within Pasifika communities, because co-design approaches often develop social change initiatives to address important issues that are relevant to peoples lived experiences. - CBPR aligns with the call for public health to better integrate research and practice, increase community involvement, partnerships, and organisation, include more holistic, partnership-based research methods, and account for cultural provisions of ethnic specific communities. | The Pasifika Prediabetes Youth Empowerment Programme research methodology and programme modules were adapted to each Pasifika context with input from the communities. The Fonofale model provided the Pasifika framework and guided how the modules incorporated Pasifika language, values and beliefs. |
| Scott 2015 | 2013 | The research team | Community members  Health providers | Community members (Samoan n=10, Tongan n=1 & Cook Islander n=1) and five Pacific health professionals. | Semi-structured interviews were conducted in 2013 with Pacific Island community members and health professionals who worked with or were connected with the Pacific community. Of the seven health professionals interviewed, three were Samoan, two Tongan and two non-Pacific islanders. At the time of the interviews, the health professionals worked in community health, primary care or tertiary hospital care. | Community engagement | No definition or explanation provided. | None stated. |
| Shintani 1994 | 1987 | The Waianae Diet Program was conceived by the principal author in mid-1987 and developed together with members of the Waianae Coast Community Committee and Waianae Coast Comprehensive Health Center staff. | Community members  Health providers  Health professionals | Members of the Waianae Coast Community Committee | The concept of the Waianae Diet Program was conceived by the principal author in mid-1987 and developed together with members of the Waianae Coast Community Committee and Waianae Coast Comprehensive Health Center staff.  The structure of Waianae Coast Comprehensive Health Center's community-based board provides for input from the community and the empowerment by the community to place resources behind problems from which the community itself suffers and wants to remedy. In late 1987, an advisory committee comprising Waianae Coast community members and health professionals of the extended Hawaiian community was formed to carry out this project. | Community-based philosophy  Empowerment | No definition or explanation provided. | None stated. |
| Simmons 2004 | Unknown | The churches.  Each of the intervention churches had invited the South Auckland Diabetes Project to work with them in developing a diabetes prevention programme through one or more church members and their church leaders. | Community organisations/service providers  Churches  Health professionals | Two Samoan and two Tongan churches | Supervision of the programme by the intervention churches was by church committees who adapted, enhanced, added to, prioritized, and co-ordinated the programmes working closely with the diabetes nurse specialist, the church members and church volunteers. Detailed tailoring occurred through language, participation of church members at all levels. Networking in the form of discussions with individual church leaders and members continued throughout the intervention and ensured that the intervention remained culturally relevant. | Community action | No definition or explanation provided. | Detailed tailoring occurred through language, participation of church members at all levels, different approaches to presentations, including humour and current affairs issues relevant to the Island group, different foods and cooking methods and different kinds of exercise, often to traditional music.  Networking in the form of discussions with individual church leaders and members continued throughout the intervention and ensured that the intervention remained culturally relevant. |
| Sinclair 2013 | Unknown | Community leaders and health advocates from four distinct community organizations serving Native Hawaiians and Pacific Islanders, and researchers from the University of Hawaii, Department of Native Hawaiian Health. | Researchers/academics  Community members  Community organisations | Native Hawaiian/Pacific Islander community members and leaders | The Partners in Care intervention was part of an existing community-based participatory research project called the PILI 'Ohana Project, comprised of community leaders and health advocates from four distinct community organizations serving Native Hawaiians and Pacific Islanders, and researchers from the University of Hawaii, Department of Native Hawaiian Health. They served as the intervention steering committee and assisted in the planning and implementation of the Partners in Care intervention. The community steering committee and focus group data informed the cultural adaptation of the intervention. To adapt the intervention, focus groups with Native Hawaiians and Pacific Islanders were conducted to provide guidance regarding the content, format, and method of delivery of the intervention. | Community-based participatory research | No definition or explanation provided. | None stated. |
| Tanjasiri 2019 | Unknown | Four Pacific Island community-based organizations and one University. | Researchers/academics  Community members  Community organisations/service providers | Leaders of Chamorro, Samoan and Tongan communities. | The entire study, from conceptualisation to completion, was guided by community-based participatory research approaches involving key personnel from four Pacific Island community-based organizations and one university.   The community advisory board provided advice throughout the study. | Community-based participatory research | No definition or explanation provided. | None stated. |

Additional files table 3: Information on interventions and effectiveness

| **Author/Year** | **Health issue** | **Intervention** | **Cultural considerations in intervention development** | **Evaluation participants** | **Data collection type** | **Main outcome** | **Sub outcomes** | **Main findings of co-designed intervention** | **Limitations** | **Article conclusions** |
| --- | --- | --- | --- | --- | --- | --- | --- | --- | --- | --- |
| Aitaoto 2012 | Breast cancer prevention | Culturally tailored educational materials and toolkit, to be delivered by lay educators.s | - Participants discussed the collectivistic orientation of Pacific Islander cultures and the expectation of reciprocity. Micronesians can be reached through church and community groups. - Women usually congregate separately from men. - Micronesians United serve as one -on-one peer educators of Micronesian women. - Involving women with a range of ages in education sessions fit well with Micronesian values of collectively and mutual support. - The Pacific manner of reciprocity and gifting also was respected. | 567 Micronesian women aged 18-75 years (202 women aged 40 or older eligible for mammography screening). | Screening data | Increase in mammograms | Lay educator skills, self-esteem, motivation to extend skills | - 11 Micronesian lay educators provided cancer information sessions to 567 Micronesian women. - Among the 202 women aged 40 or older eligible for mammography screening, 166 (82%) had never had a mammogram and were assisted to appointments. - After 6 months, 146 (88%) of the 166 had received a mammogram, increasing compliance from 18% to 90%. | Short follow-up time due to funding.  User perception of lay educators was not collected  Outreach took longer than expected. | The CBPR process was useful in developing lay navigator training and materials for this unique community.  The Micronesians United champion was a founding member of the group who was a trusted advocate for Micronesian issues. Their leadership of the weekly meetings ensured a safe forum for lay educators to discuss successes and concerns, solve problems, and translate their new knowledge and skills into practice with remarkable speed. |
| Chung-Do 2024 | Food insecurity | Seven workshops: Indigenous knowledge, gardening and learning how to build and maintain home backyard aquaponics.  These workshops also embraced the Indigenous knowledge that “food is medicine” by integrating culturally grounded teachings, such as ‘ai pono (healthy eating) and lā’au lapa’au (traditional spiritual medicine). | - To be mindful of Native Hawaiian collectivistic values, children under the age of 18 were invited to participate with adult family members in the workshops but were not considered as research participants. - To promote cultural relevance and a family-friendly setting, meals were provided as well as a hands-on cultural activity. | 21 participants from 10 Native Hawaiian families enrolled in the MALAMA study (18-68 years). | Pre & post anthropometric/metabolic data, survey data and focus groups | Eating habits | Blood pressure, waist-to-hip ratio and BMI | - Fruit consumption among all participants significantly increased from time 1 (2.1 servings) to time 2 (2.9 servings). - No statistically significant differences in body mass index and waist-to-hip ratio were observed between any time points among participants, although there were favourable trends in blood pressure and fish and vegetable consumption. | Small sample size  Short follow-up timeframe | Community-driven and culturally-grounded solutions, such as MALAMA, may be a promising approach to addressing pervasive health disparities and promoting health equity in minority and Indigenous communities. |
| Firestone 2021 | Diabetes prevention | Eight-week programme with education on physical activity, nutrition and building health literacy. | Unknown | Pacific and Māori aged between 25-44 years old with a high risk of developing prediabetes | Pre & post anthropometric and physical activity data, interviews | Daily physical activity (measured by bodyweight loss and increased step counts) | User engagement & understanding, sustainability | Significant positive changes, as evident by the mean percent change in weight loss (2.43%), mean percent change in waist circumference reduction (1.58%) and total average number of steps (range: 14,817-80,182 steps) accumulated from the start of the intervention (p<0.001). | Small sample size  Short timeframe to implement intervention  Lack of research protocol ensuring youth/community facilitators recorded data completely. | The success of the intervention was based on the co-designed approach of the study.  The achievement of more than 2% weight loss over a short period of time is a strength compared to longer studies, and this is indicative of the capability of the youth and community facilitators to motivate behavioural change. |
| Fitzpatrick 2007 | Malaria prevention | Distribution of insecticide impregnated bed nets. | This model of health promotion emphasised that the relevance, timing, venue content and style of delivery should match the cultural experience of the participants. Education sessions about care and maintenance of the bed nets were conducted by an author on the Batri airstrip. | Community members in Batri village | Anecdotal evidence, mortality data | Malaria-related deaths | N/A | Records prior to November 2004 at the remote Batri Village Aid Post showed that, among the village population of 1400, there were five deaths from malaria each year and 29 consultations for malaria-related symptoms. Since the deployment of the nets in Nov 2004 there were no deaths from cerebral malaria in the community. | None stated. | The project design accommodated the logistics of using a partnership approach to work across diverse cultures. What emerged was that a genuine interest in, and affirmation of, the local culture is the crucial factor in a positive outcome. |
| Fotu 2011 | Obesity | Social marketing, community capacity building and grass roots activities to promote healthy behaviours. | Unknown | 1083 adolescents in intervention communities. 1396 adolescents in the comparison group. | Pre and post data (metabolic, behavioural changes) | Body fat % | BMI, BMI-z | - No statistically significant differences in outcomes in weight, BMI and BMI-z, or prevalence of overweight/obesity between the intervention and comparison groups. - A small relative decrease in body fat in the intervention group (-1.5%, P < 0.0001). No other difference for any anthropometric variables between groups. | Although socio-cultural interviews were conducted prior to the intervention, the short lead-in time meant that factors could not be fully integrated into the intervention. | The Ma’alahi Youth Project had no impact on the large increase in prevalence of overweight and obesity among Tongan adolescents.  The decrease in percentage body fat was a promising shift; however, the lack of change in BMI and BMI-z and most behaviours suggests the intervention was insufficient to slow unhealthy weight gain. |
| Han 2015 | Mental health and wellbeing | Community organising campaign led and run by Pacific youth | Unknown | Pacific youths | Pre and post surveys, focus groups, interviews | Mental health outcomes | Personal agency | - Qualitative and quantitative data showed that youths experienced improvements in their mental health status. - Data from the surveys (p<0.1) showed that youths felt calmer and more relaxed, reporting being more likely to think before acting and less likely to experience headaches, stomach aches or sickness. | Low numbers of respondents  Short time horizon | CM Health equipped youths with the capacities they needed to make changes. Although these pilot data are limited by the low numbers, initial results show that organising has potential as a preventive approach to improving mental health. |
| Kaholokula 2017 | Hypertension | 12-week hula-based intervention with education on blood pressure management.  This was a pilot study using a 2-arm RCT with a wait-list control group. | Hula is the traditional dance of Native Hawaiians. It is performed by men and women of all ages. Originally performed to convey history, spiritual beliefs, and one’s connection to the natural world, hula is now practiced as a form of cultural and creative expression.  The hula training promotes the Hawaiian value of interconnectedness between individuals and fosters a family-like environment. | 55 participants with diagnosed hypertension | Metabolic data | Blood pressure | Physical functioning (measured by 6min walk test), health-related quality of life | - Ola Hou participants, compared to wait-list control participants, had greater reductions in their systolic blood pressure in both the intention-to-treat analysis (-18.3 vs -7.6 mmHg, respectively) and the complete case analysis (-19.8 vs. -9.2 mmHg, respectively) from baseline to 3-month assessment. - No significant differences in diastolic blood pressure between the intervention and control after 3 months. | Subjective self-reported measures used  Lack of data on hypertension medication information | The hula-based Ola Hou program led to significantly greater reductions in SBP and notable improvements in bodily pain when compared to those randomized to the wait-list intervention control group. However, there were no statistically significant differences between the intervention and control group on measures of physical functioning and HRQL. |
| Katz 2007 | HIV/AIDS | Tingim Lap intervention: peer education, community mobilisation, life skills development and sports/music interventions. | A bottom-up approach was taken to communication priorities, strategies and messages. Local communities develop messages according to local needs, realities, interests, customs and languages with technical support provided as required. This approach helps to ensure that local meaning is given to messages helping to support attitudinal, behavioural and social change. | PNG people living with HIV/AIDS and youth | Anecdotal evidence, observational data | Attitudinal and behavioural change to prevent HIV | Increased voluntary testing and counselling, increased demand for condoms | - 34 Tingim Laip sites around PNG reached an estimated at +170,000 persons. - Qualitative data showed improvements as a result of the intervention and local STI services in most of the sites reported increased number of clients utilizing their services as a result of the demand generated by the intervention. | None stated. | Whist Tingim Laip is a young intervention, it is already showing promising signs of playing an important role in bringing about attitudinal, behavioural, and social change required to tackle the HIV epidemic in especially vulnerable  parts of PNG. |
| Kaufer 2010 | Non-communicable diseases | Two-year food-based intervention focused on education, social marketing and agricultural activities e.g. providing planting materials and training workshops. | This participatory, inter-agency, community-based approach used education, social marketing and agriculture to increase local food production and consumption, while also incorporating concepts of local culture, environmental sustainability and long-term food security. | Random sample of households (n=47) out of all 71 households in Mand. | Pre & post surveys, dietary assessments, interviews | Dietary changes | Attitudes toward local food | - The average household diet in 2007 (follow-up) had significantly higher micronutrient intake, increased consumption frequency of promoted foods and greater dietary diversity than in 2005 (baseline). - Results indicated increased (110%) provitamin A carotenoid intake; increased frequency of consumption of local banana (53%), giant swamp taro (475%), and local vegetables (130%); and increased dietary diversity from local food. | Reduced internal validity due to influence of external events, maturation of the subjects and the educational effect of the pretest.   Short observation period. | The intervention approaches appear to have been successful in this short period. It is likely that similar approaches in additional communities in Pohnpei and other parts of the Pacific would also be successful in promoting local food. |
| Kremer 2011 | Obesity | Social marking, nutrition and physical activity initiatives. | Unknown | 7 intervention schools (n=874) and 11 comparison schools (n=2,062). Participants were aged 13-18 years. | Pre and post surveys, anthropometric data | BMI & body fat % | Quality of life, behaviour change | - At follow-up, no differences for weight, body size and weight status classification. The intervention group had lower percentage body fat (-1.17). - The (unadjusted) proportion of children who reported having snack food every day after school was lower at follow-up for both groups. A positive (healthful) change in time spent watching TV was also observed. | Short duration period  Did not address sociocultural factors, cultural values and expectations. | Overall, the findings suggest that this school-based health promotion programme did not achieve the desired goals of reducing unhealthy weight gain or attenuating some obesity-promoting behaviours.   Additional top-down or other innovative approaches may be needed to reduce adolescent obesity in the Pacific. |
| McElfish 2015 | Diabetes | 10 hours of diabetes education over six weekly sessions.  The topics included: healthy eating, being active, glucose monitoring, understanding blood glucose and taking medication, problem solving, reducing risks and healthy coping, mitigating complications of diabetes, and goal setting. | Matriarchal kinship play a key role in the life of the Marshallese patient with diabetes. The Marshallese community suggested that for the individual to change, the family must change.  Thus diabetes education should be implemented within an extended family model so that the entire family could benefit from the education, and the patient could be supported. | Six Marshallese families (27 participants). | Pre and post test design, questionnaires, biometric data | Glycemic control measured by A1C | Total cholesterol, HDL, BMI | 78% of participants were retained in the study. Post-test results indicated a 5% reduction in A1C across all participants and a 7% reduction in those with type 2 diabetes. | Small sample size  Varying levels of family member engagement in intervention | Building on the emerging literature of family models of diabetes self-management education, this study shows that the family model delivered in the home had high acceptance and that the intervention was more accessible for this hard-to-reach population. |
| McElfish 2019 | Glucose monitoring for type 2 diabetes | Health education video highlighting the importance of performing blood glucose checks. | The video was produced in Marshallese with English subtitles, using plain language for those with low health literacy.  Marshallese community members, health workers and researchers collaboratively edited the video for both medical accuracy and cultural appropriateness. | 50 Marshallese participants with type 2 diabetes mellitus (20 of which completed semi-structured interviews). | Pre & post surveys, semi structured interview | Participants' self-efficacy related to glucometer usage | Cultural appropriateness and effectiveness of video | - Participants reported significant increases in self-efficacy related to glucometer use and the importance of performing blood glucose checks (p <.001) and a 1.45% reduction in A1C between preintervention and 12 weeks postintervention (p=.006). - Qualitative results indicated the video was both culturally appropriate and effective. | Small sample size  Recruitment from one single clinic  No control group | Using a community-based participatory research approach to prioritize video topics and including members of the community in the creation and dissemination of the videos, could aid in ensuring the videos are effective and culturally appropriate. |
| Mishra 2009 | Cervical cancer education | Cervical cancer education programme: education booklets, skill building, interactive group discussion sessions. | Trained Samoan lay health educators delivered the education program and materials were written in the Samoan language.  The booklet presented the content in culturally appropriate and acceptable language, giving due deference to the religious and traditional, including sensitive information about the female anatomy and sex-related risk factors. | 416 eligible women recruited from the 26 Samoan churches in the two study locations | Pre & post surveys | Utilisation of Pap smears | Knowledge of cervical cancer, self-efficacy | 120 women (30.2%) self-reported obtaining a Pap smear between the pretest and posttest surveys. Women in the intervention group (61.7%, n=74, p < 0.01) compared to those in the control group (38.3%, n=46) were significantly more likely to self-report having obtained a Pap smear. | Data is self-reported | In conclusion, findings from this study suggest that a multifaceted, theoretically-guided, culturally tailored cervical cancer education intervention can improve Pap smear use among Samoan women and effect positive changes in knowledge and attitudes. |
| Ndwiga 2020 | Type 2 diabetes | Church-based lifestyle education and support programme delivered by Community Coach Facilitators and Peer Support Facilitators to prevent and promote self-management of Type 2 diabetes. | Two University employed Samoan Coach Facilitators who could speak and write fluently in both English and Samoan alongside volunteer Peer Support Facilitators delivered the intervention.  The churches took on many key messages e.g. introducing healthier foods during Sabbath feasting. | Three churches took part. 107/159 (67%) church attendees participated in the baseline. | Pre and post questionnaires, anthropometric data, interviews | HbA1c | BMI, physical activity, diabetes knowledge | - HbA1c dropped significantly between baseline and follow-up among participants with known diabetes (8.1 ± 2.4% (65 mmol/mol) vs 7.4 ± 1.8% (57 mmol/mol); p = 0.040) and non-significantly among participants with newly diagnosed diabetes (8.0 ± 2.1% (64 mmol/mol) vs 7.1 ± 2.3 (54 mmol/mol); p = 0.131). - There were no significant reductions in blood pressure, BMI or waist circumference at follow-up. | Small sample size  Lack of control group  Limited duration | A structured, church-based, culturally tailored lifestyle intervention showed a number of improvements in diabetes risk among Samoans in Sydney. The intervention however, requires a more rigorous testing in a larger randomised controlled trial over a longer time period. |
| Oliver 2007 | Type 2 diabetes | Lifestyle interventions: Walking groups, line dancing, gym work, food diaries and a nutrition workshop. | Planned activities that respected cultural and socio-economic factors were implemented. | The same 24 Cook Islands participants who were involved in developing the interventions. | Focus groups, self reported/anecdotal data | Physical activity, nutrition | Awareness of health, weight loss | - After six weeks, members of the walking groups were still walking regularly, some having increased activity from three times per week to daily. - Some women had lost a considerable amount of weight, which motivated others to increase their exercise levels. | Focus groups were time limited.  Changing beliefs and attitudes takes time. | The study shows that action research methods can be used effectively to influence behaviour changes in small, culturally and linguistically diverse communities. |
| Prapaveissis 2022 | Prediabetes | Pasifika Prediabetes Youth Empowerment Programme: Seven empowerment modules and five co-design modules | Focus group discussions are a culturally appropriate method of data collection for peoples that value collectivism. Focus group discussions draw parallels to talanoa.  Within a Pasifika research setting, community individualisation must encompass cultural provisions and beliefs and this model provided opportunities to account for the unique realities of each community context. | 41 Pacific youth participants in total started the programme and 29 were retained over time. | Open-ended evaluation surveys, semi structured focus groups, key informant interviews | Knowledge about healthy lifestyles | Building leadership and social change capacity | - The programme increased youth's knowledge about health and healthy lifestyles, developed their leadership and social change capacities, and provided a tool to develop and refine culturally centred prediabetes-prevention programmes. - The programme increased the youth's awareness about the intersectionality between mental wellness and obesity, prediabetes and Type 2 diabetes for Pasifika. | Volunteer sample selection bias  Attrition bias | Empowerment and co-design are effective tools to develop and implement culturally tailored health promotion programmes for Pasifika peoples. Future research is needed to explore the programme within different Pasifika contexts, health issues and Indigenous groups. |
| Scott 2015 | Immunizations | Catch-up immunisation clinics at a Samoan church. | Two immunisation catch-up clinics were held in a large Samoan church in western Sydney. The clinics were advertised through a flyer and word of mouth. The first clinic was held on a rehearsal day for a Samoan festival, which was suggested by the church leader, because many children and youth would be present. | Attendees of a Samoan church in Western Sydney aged 7-33 years | Semi structured interviews, immunization data | Immunization uptake | N/A | Approx 70 children and youth were in attendance at the first clinic held on a festival rehearsal day. There were 63 participants at the first clinic and 15 at the second. Of these, 27 at the first clinic and one at the second clinic were appropriately vaccinated; a total of 50 doses of vaccine were provided: 36 at the first clinic and 14 at the second. | Small numbers of interviewees | The outcomes of the interviews and the subsequent clinics highlighted the potential of churches as a venue for providing public health interventions such as catch-up immunization |
| Shintani 1994 | Obesity and chronic disease | Waianae Diet Program: Three week programme of traditional Hawaiian diet and cultural teachings. | ﻿The program was taught in a culturally appropriate way by the kupuna (Hawaiian elders) and other teachers of Hawaiian culture. Topics such as la'au lapa'au (Hawaiian herbal medicine) and lomilomi (Hawaiian massage) are covered.   For example Kalo (taro) in Hawaiian beliefs is the first born child of Papa (Mother Earth) and Wakea (Father Heaven) and is the eldest sibling of the human race.   Social support is an essential part, creating the essence of family or ohana. | 120 individuals in Waianae, Hawaii have gone through the program since inception. | Metabolic data | Weight | Blood pressure, serum lipids, serum glucose | - The program demonstrated significant weight loss with no calorie restriction, improvement in blood pressure, serum glucose, and serum lipids, with wide acceptance. - After 3 weeks, the 21 participants lost an average of 17.1 pounds (p<0.0001). Caloric intake decreased about 40%. Total serum cholesterol levels decreased significantly with an average decrease of 14.1%. Glucose levels reduced by 23.8% on average (p<0.01). Systolic blood pressure decreased by 8.6% (p<0.01) and diastolic by 10.6% (p<0.01). | None stated. | The WDP was effective in generating interest in diet and lifestyle change among members of a high risk, hard to reach, special population - the Native Hawaiians. Short term results are significant in reducing weight, blood pressure, serum lipids and serum glucose. |
| Simmons 2004 | Diabetes | Modular lifestyle and diabetes awareness intervention. | In South Auckland, New Zealand, an area with a high proportion of Pacific Islands people, we established a church-based diabetes prevention programme  Both churches used leaflets in their first languages and a specially designed video in English (translated piecemeal by the South Auckland Diabetes Project church member | 222 members from Samoan Seventh Day Adventist  294 members from Tonga Latter Day Saints. | Questionnaires | Weight | Diabetes knowledge, regular exercise, readiness to change weight | - In one intervention church, weight gain was controlled (vs. control 0± 4.8 vs.+3.1 ± 9.8 kg, respectively; P = 0.05), diabetes knowledge (+46 ± 26% vs.+4 ± 17%; P < 0.001) and regular exercise (at least 3 days per week: +22% vs. 8%; P = 0.032) increased. - The other intervention church increased diabetes knowledge (+19 ± 24 vs. +8 ± 25; P < 0.024), but no other significant changes. | Complexity of church structures limited monitoring | A moderate intensity, community-based, structured diabetes awareness and lifestyle programme can reduce diabetes risk. Continuous and detailed monitoring of penetration of interventions may be essential to help guide the timing of interventions and identify the need for additional strategies to increase participation and motivation. |
| Sinclair 2013 | Diabetes self management | Partners in Care intervention: Diabetes self-management education intervention. | The peer educators and steering committee members contributed local and cultural knowledge for the intervention by reviewing materials.  Images of Hawai'i, local foods, physical activities, and people were included to convey relevance to participants. Peer educators from the participating communities delivered the intervention and used “local” language. | 82 participants (48 assigned to intervention and 34 waitlist control). | In-person interviews | HbA1c | Diabetes self-management understanding, performance of self-care activities, and diabetes-related distress. | Significant baseline adjusted differences at 3 months between the Partners in Care intervention and waitlist control group in intent-to-treat (p<0.001) and complete case analyses (p<0.0001) for A1c, understanding (p<0.0001), and performing diabetes self-management (p<0.0001). | Attrition rate higher among youth | A culturally adapted diabetes self-management intervention of short duration was an effective approach to improving glycemic control among Native Hawaiian and Pacific Islanders. |
| Tanjasiri 2019 | Cervical cancer screening | A single session educational intervention to increase men’s social support for their female wives/partners to receive a Pap test and for women to receive a Pap test. | Educational approaches were discussed among community partners to identify community- and culture-specific issues relating to discussing women's health (such as strict taboos against mixed gender discussion for Tongans); spirituality and humour; and language translation needs. Education sessions were held at convenient meeting places and times, such as in the evenings at churches and community centers. Sessions always involved food and lasted approximately 2 hours. | Women: 591 total; 249 intervention and 342 control Men: 416 total; 150 intervention and 266 control | Pre and post surveys | Uptake of Pap test in women. | Women's pap testing knowledge and attitudes, support from male partners | - Intervention women who were not compliant with Pap screening recommendations at pretest were significantly more likely to have scheduled and received a Pap test at 6-month follow-up (Beta 0.820 (SE 0.45), 95% CI -0.071, 1.171). - However, 6-month follow-up results indicated no intervention effect on changes in women's Pap testing knowledge (p=0.64), fatalistic attitudes (p=0.454), or perceived social support from their male partner (p=0.36). | Self-reported data  Loss to follow-up | Ethnic- and gender-tailored community interventions can successfully increase Pap test behaviours for PI women, although more research is needed on the specific pathways leading to behaviour change.  Impact: collaborative community-based interventions lead to increases in women's cancer prevention and early detection for Pacific Islander and other collectivistic communities |
