# Supplementary material for: The processes and impacts of co-designed health interventions by and for Pacific populations: a scoping review
Source: BMC Public Health. 2025 Jul 26;25:2555. doi: 10.1186/s12889-025-23795-w (PMC12297805; doi:10.1186/s12889-025-23795-w)
Supplement: Supplementary file 4 — Additional file 4. Data extraction tables [file 12889_2025_23795_MOESM4_ESM.docx]

## **Additional files 2: Search strategy**

Electronic databases


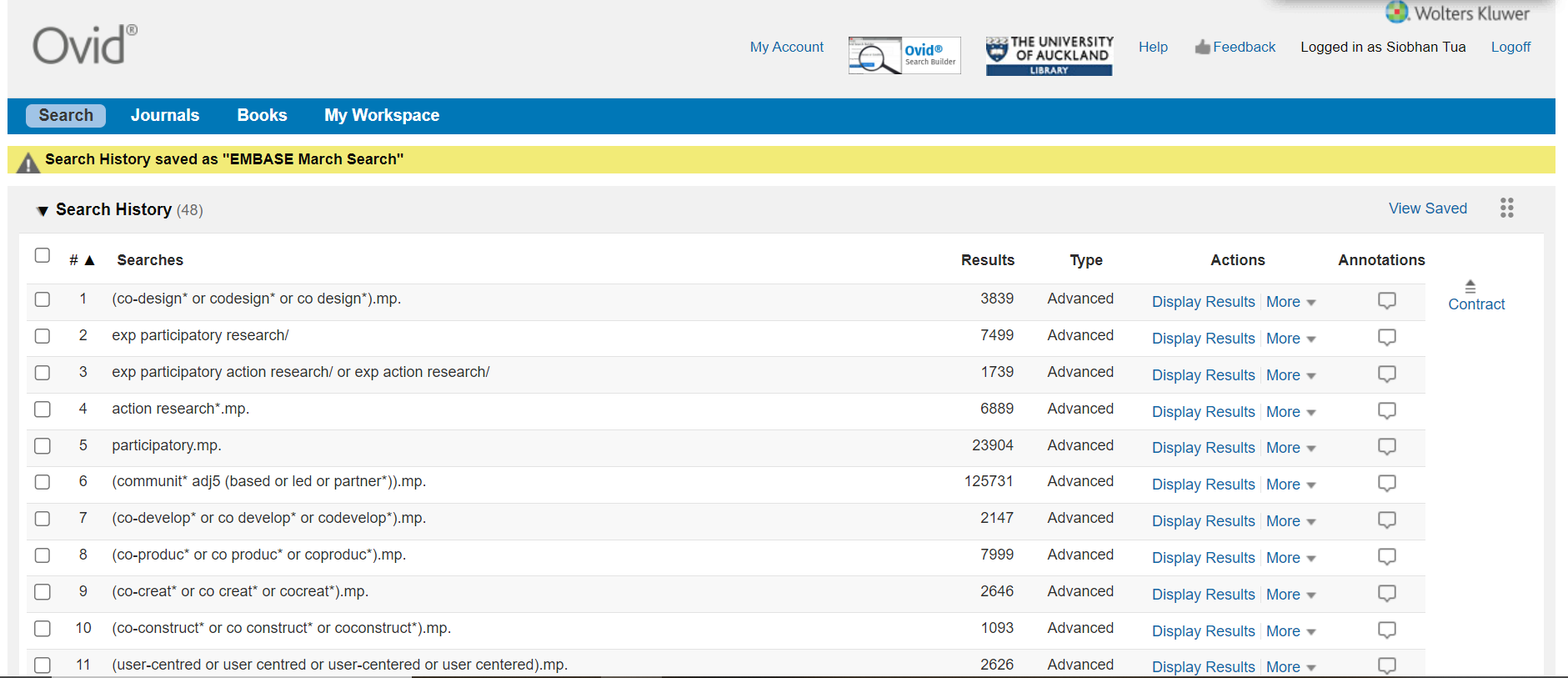
EMBASE via Ovid


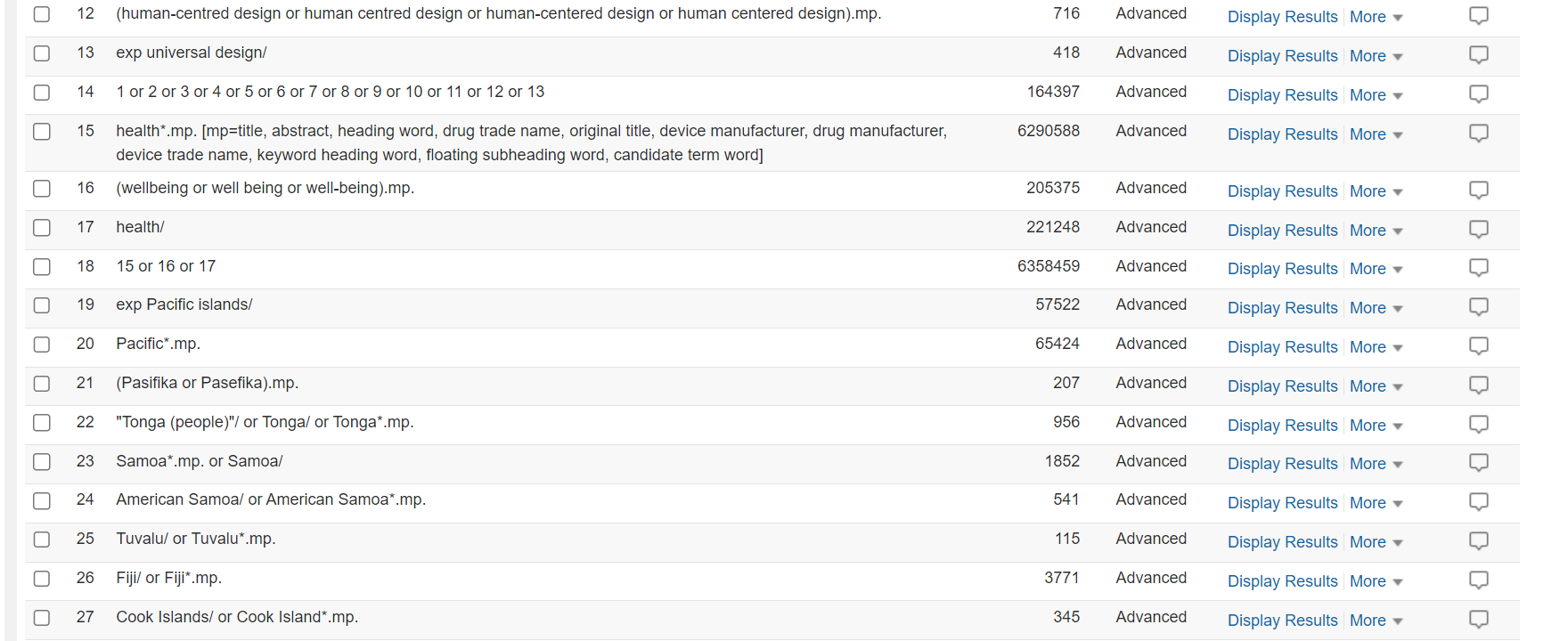


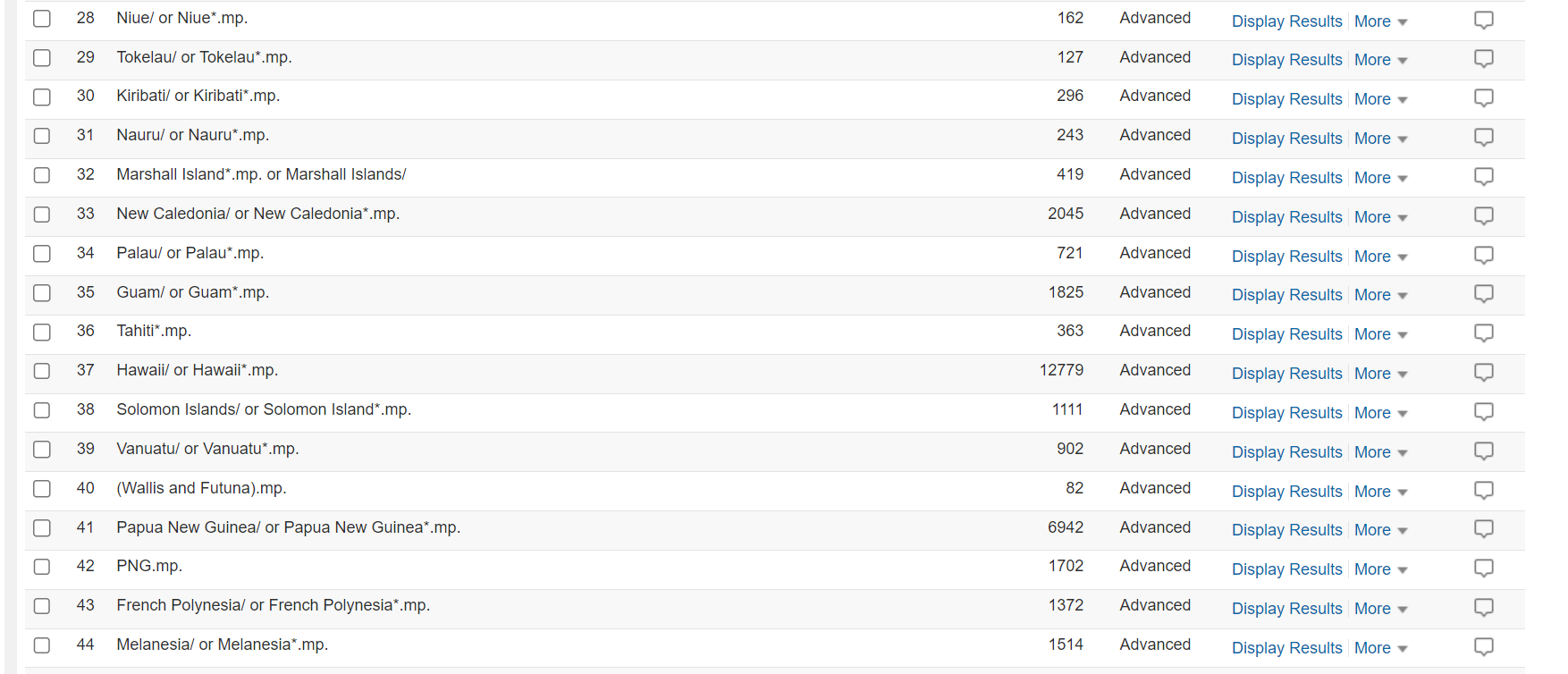


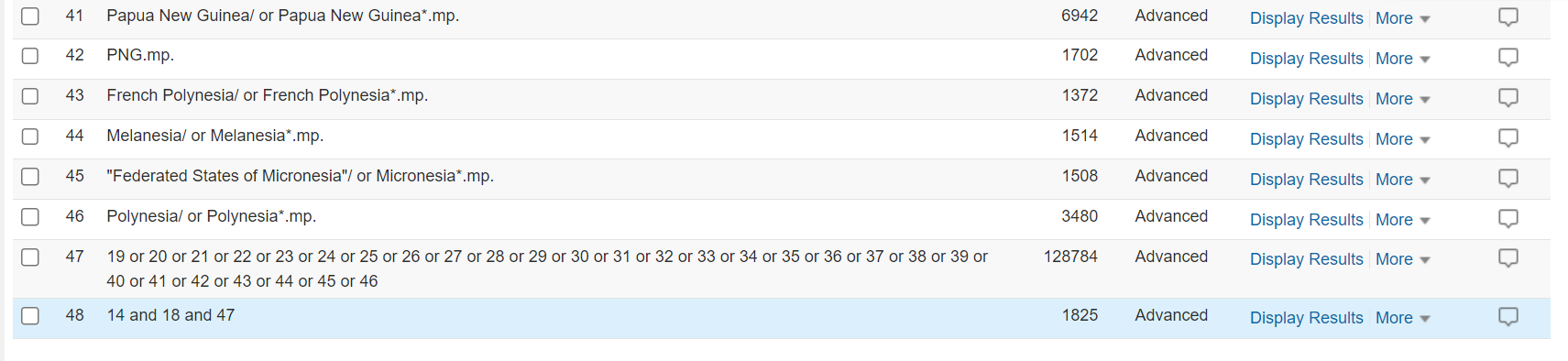


MEDLINE via Ovid


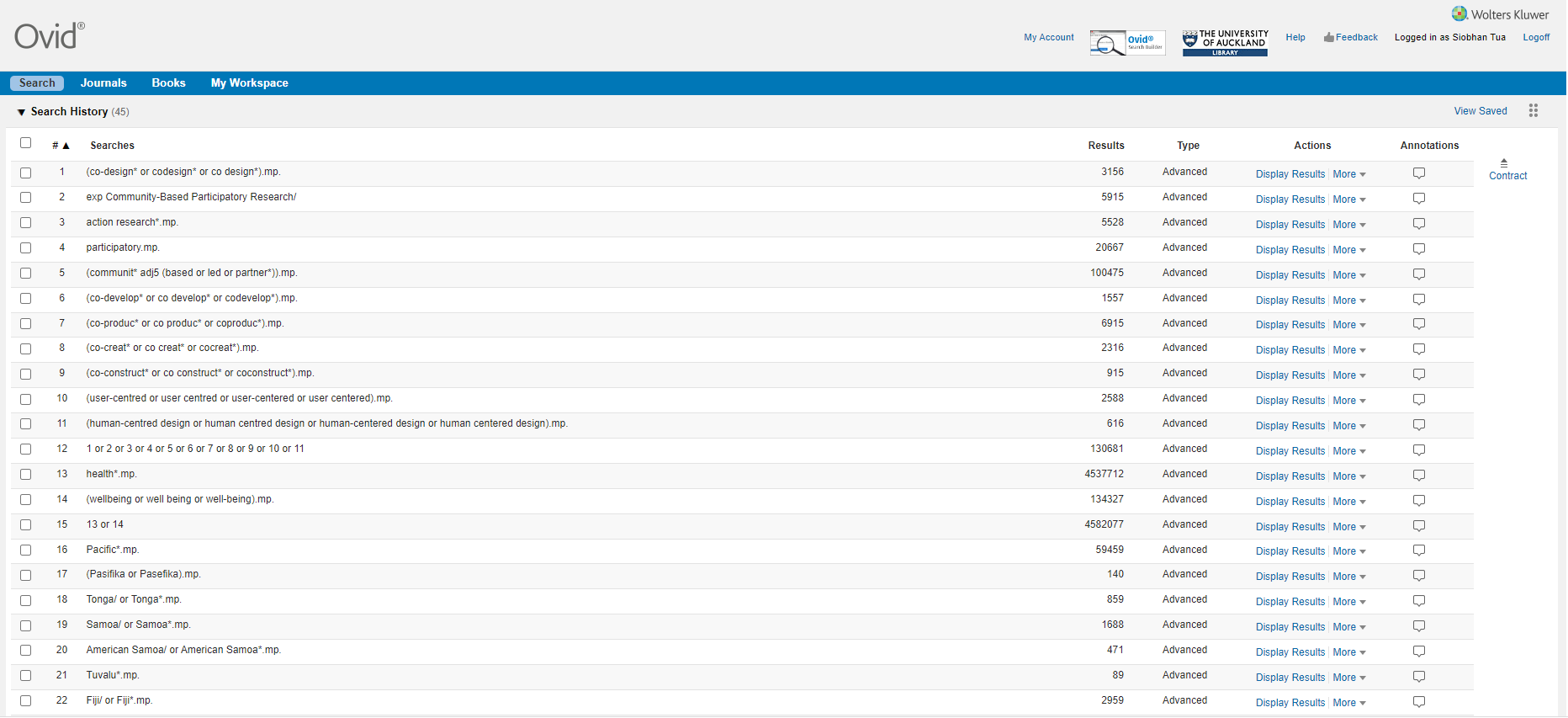


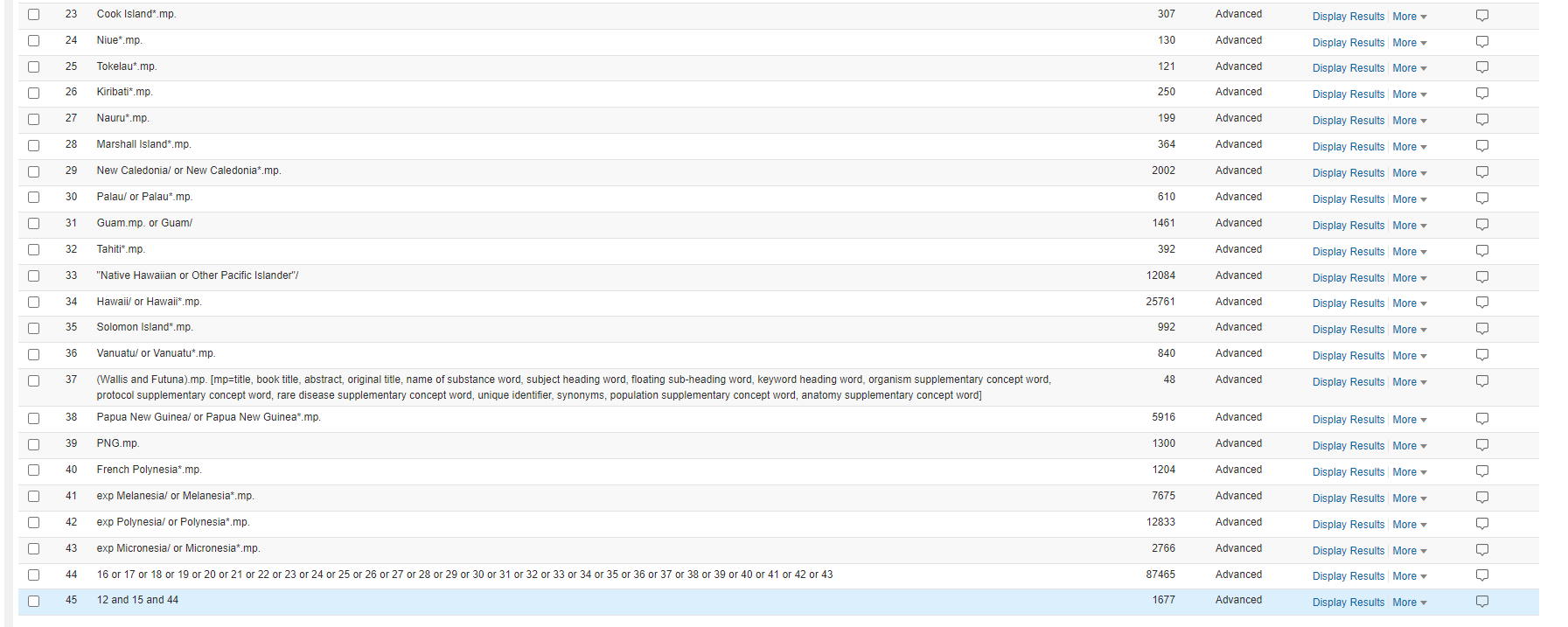


PsycINFO


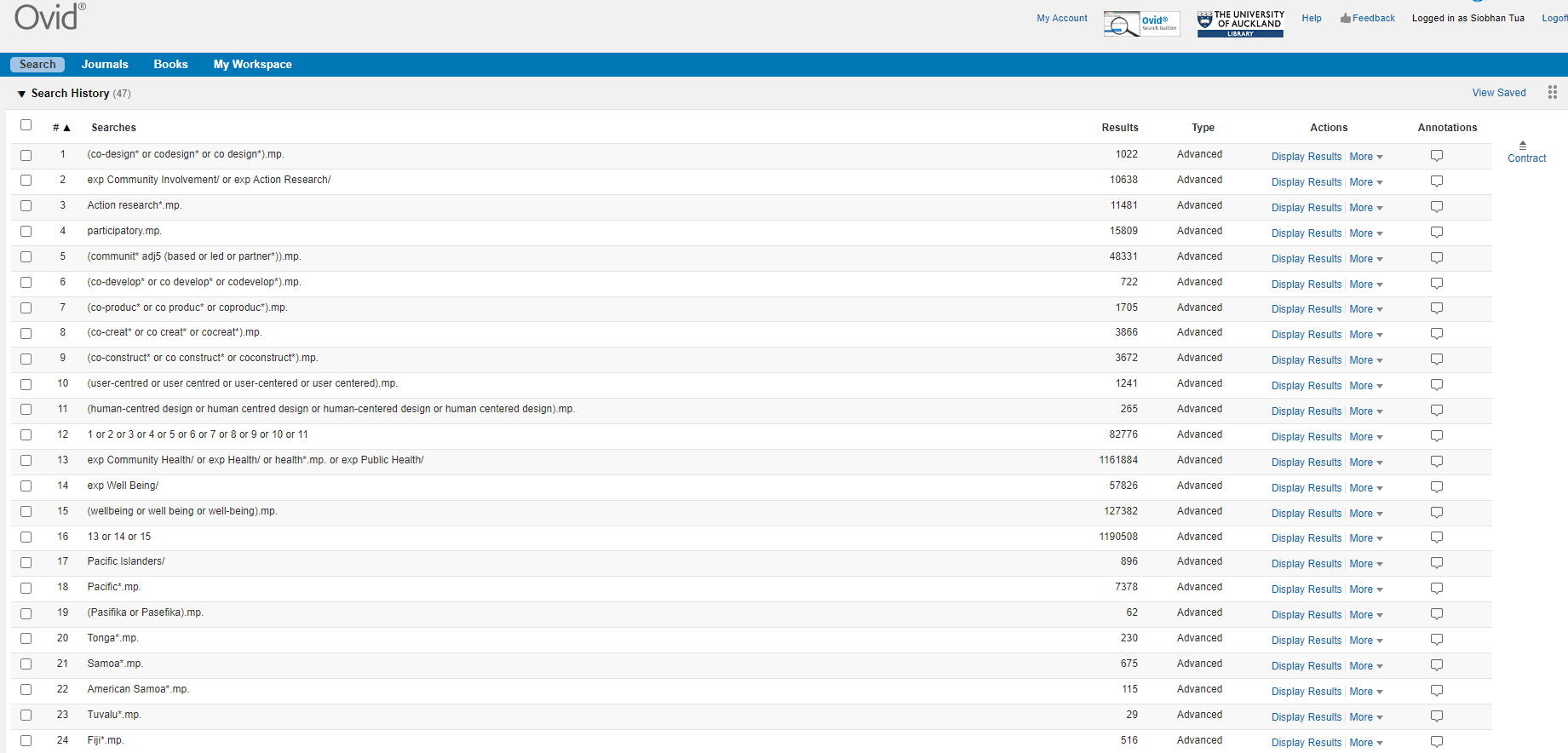


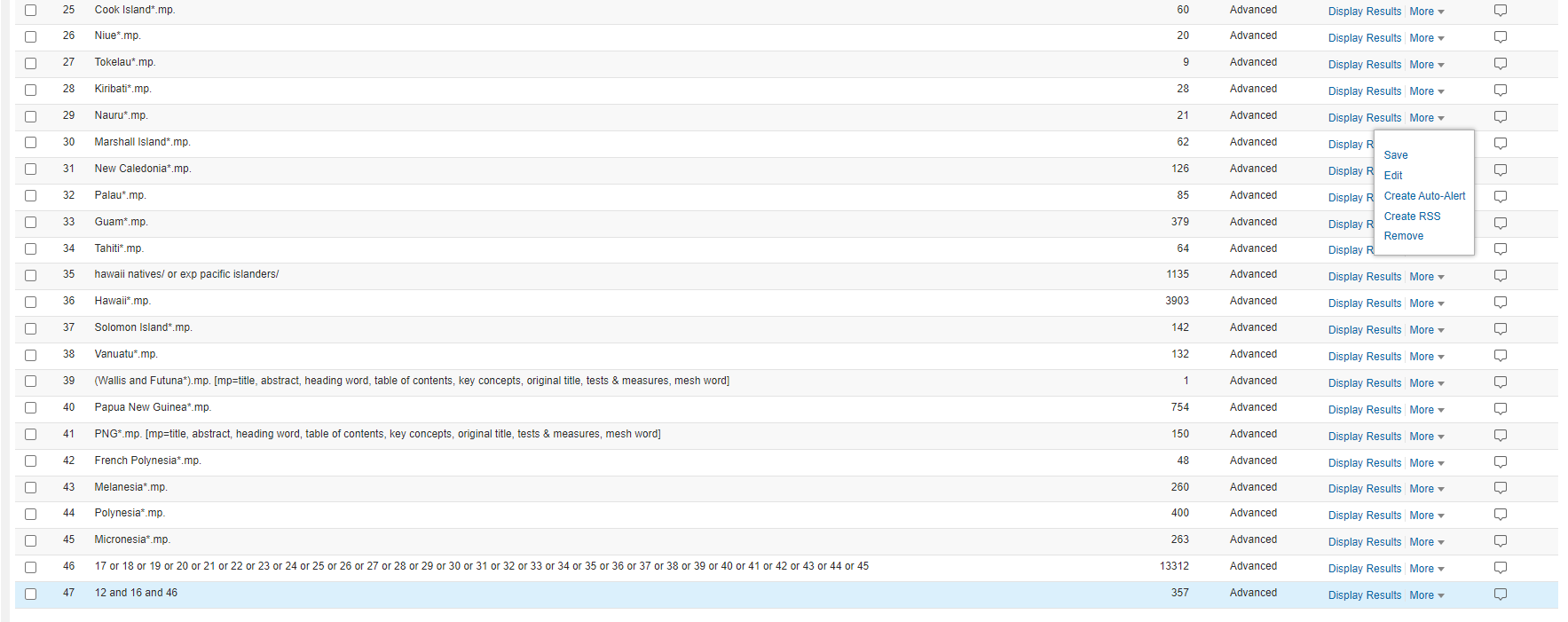


Scopus


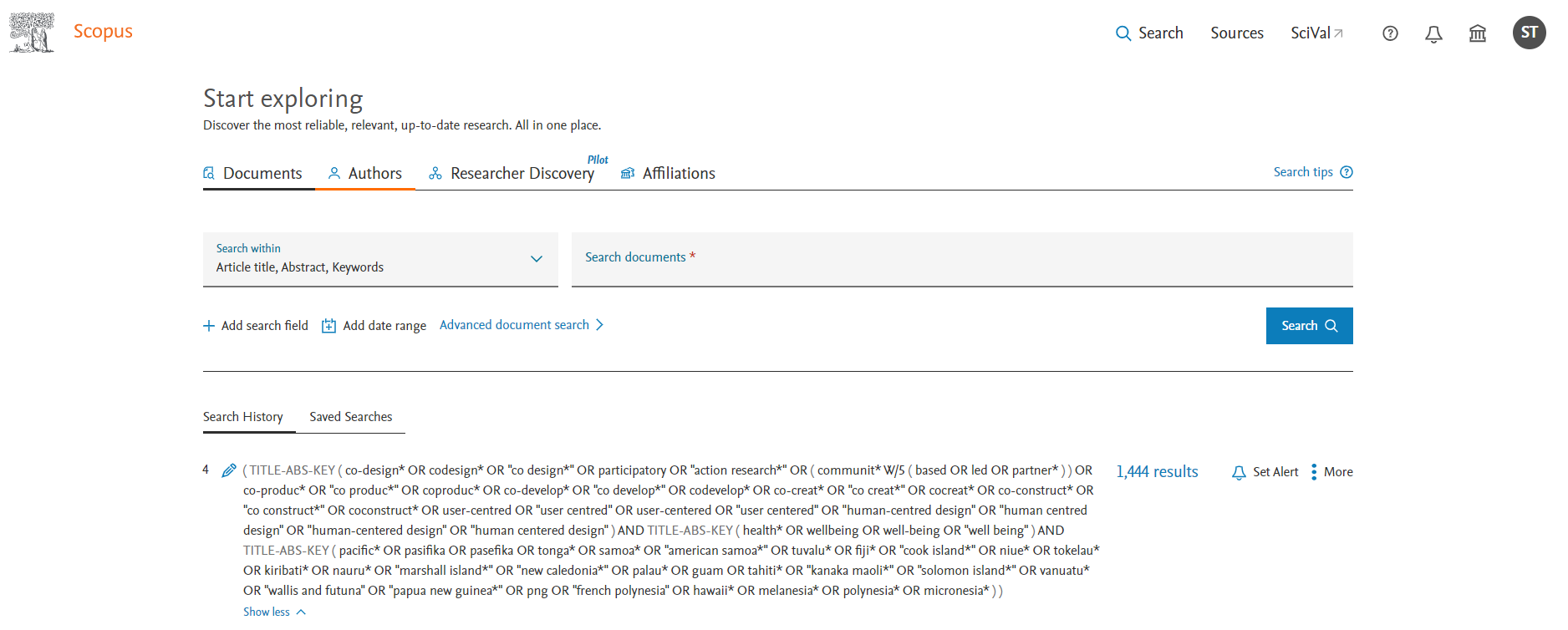


CINAHL


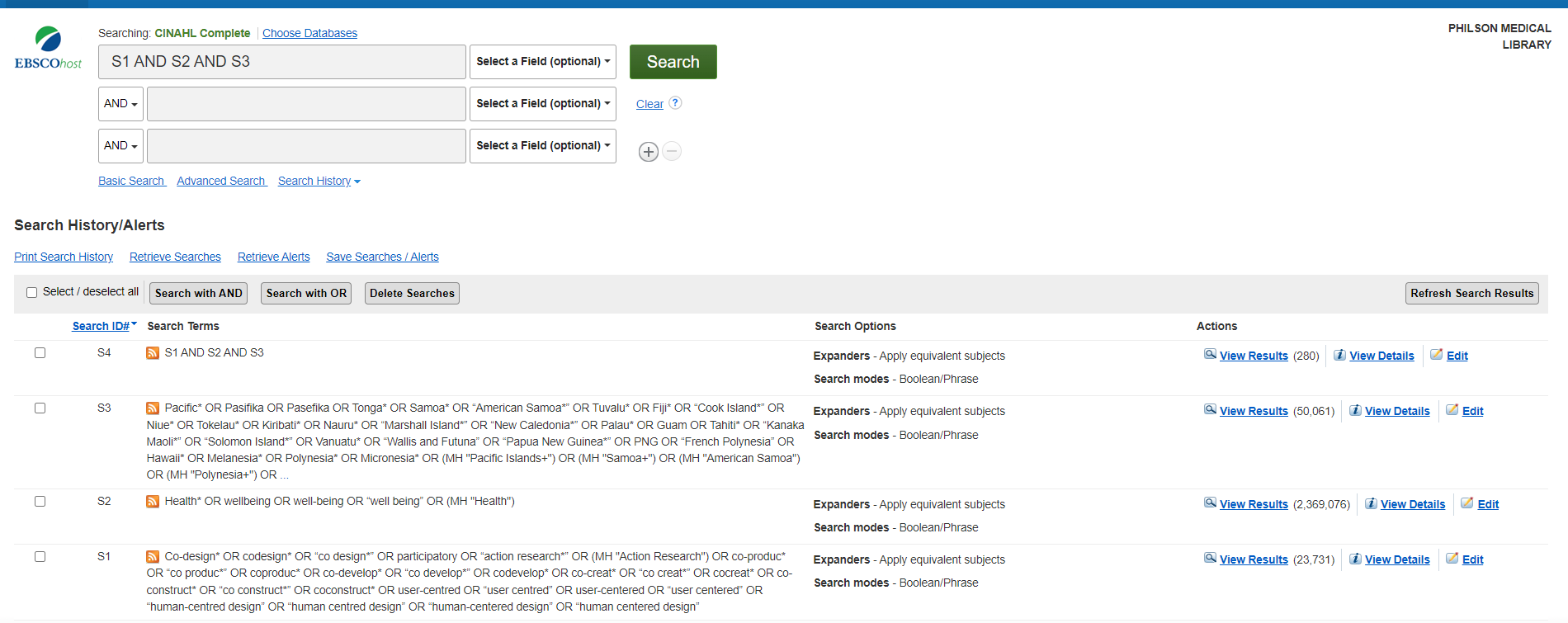


Note: Updated searches were re-run on 27 January 2025. A total of 1001 new records (since the initial search) were found across the above databases.

Grey literature

Searches were conducted for governmental websites, health ministry reports and thesis repositories on 12^th^ May 2025.

- Governmental and health ministry websites
  - American Samoa <https://doh.as/>
  - Australia <http://health.gov.au/>
  - Cook Islands <https://www.health.gov.ck>
  - Federated States of Micronesia <https://hsa.gov.fm/>
  - Fiji <https://www.health.gov.fj/>
  - Guam <https://opa.hhs.gov/>
  - Hawaii <https://health.hawaii.gov/>
  - Kiribati <https://mhms.gov.ki/>
  - Marshall Islands <https://rmihealth.org/>
  - Nauru <http://naurugov.nr/government/departments/department-of-health-and-medicinal-service.aspx>
  - New Caledonia <https://dass.gouv.nc/>
  - New Zealand <https://www.tewhatuora.govt.nz/> and <https://www.health.govt.nz/>
  - Palau <https://www.palaugov.pw/executive-branch/ministries/health/>
  - Papua New Guinea <https://www.health.gov.pg/index.html>
  - Samoa <https://www.health.gov.ws/>
  - Solomon Islands <https://solomons.gov.sb/ministry-of-health-medical-services/>
  - Tahiti/French Polynesia <https://www.cor-ntd.org/research-outcomes/institutions/french-polynesia-ministry-health-moh>
  - Tokelau [www.tokelau.org.nz/Tokelau+Government/Government+Departments/Department+of+Health](http://www.tokelau.org.nz/Tokelau+Government/Government+Departments/Department+of+Health)
  - Tonga <https://www.health.gov.to/node/391>
  - Tuvalu - <https://tuvalu.tradeportal.org/?l=en>
  - United States US Department of Health and Human Services <https://www.hhs.gov/>
  - Vanuatu <https://moh.gov.vu/>
  - Wallis and Futuna – no website available
- Organisations
  - World Health Organization <https://www.who.int/>
  - LeVa <https://www.leva.co.nz/>
  - Moana Connect <https://moanaconnect.co.nz>
  - Pacific Community (SPC) <https://php.spc.int/>
- Thesis repositories
  - <https://www.proquest.com/>
  - <https://digitalnz.org/nzresearch>
- Google search (first 50 results)
